# Low-dose atrial natriuretic peptide for prevention or treatment of acute kidney injury: a systematic review and meta-analysis

**DOI:** 10.1186/s13054-019-2330-z

**Published:** 2019-02-11

**Authors:** Hiroyuki Yamada, Kent Doi, Tatsuo Tsukamoto, Hideyasu Kiyomoto, Kazuto Yamashita, Motoko Yanagita, Yoshio Terada, Kiyoshi Mori

**Affiliations:** 1 Department of Nephrology, Graduate School of Medicine, Kyoto University, 54 Shogoin-Kawahara-cho, Sakyo-ku, Kyoto, 606-8507 Japan; 2 Department of Emergency and Critical Care Medicine, The University of Tokyo, Tokyo, Japan; 3 Department of Nephrology and Dialysis, Kitano Hospital, Tazuke Kofukai Medical Research Institute, Osaka, Japan; 4 Division of Integrated Nephrology and Telemedicine, Department of Community Support, Tohoku Medical Megabank Organization, Tohoku University, Sendai, Japan; 5 Department of Healthcare Economics and Quality Management, Graduate School of Medicine, Kyoto University, Kyoto, Japan; 6 Department of Endocrinology, Metabolism and Nephrology, Kochi Medical School, Kochi University, Nankoku, Japan; 7 Department of Nephrology and Kidney Research, Center for Public Health, Shizuoka General Hospital, Shizuoka, Japan; 8 Department of Molecular and Clinical Pharmacology, School of Pharmaceutical Sciences, University of Shizuoka, Shizuoka, Japan

**Keywords:** Acute kidney injury, Atrial natriuretic peptide, Carperitide, hANP, Systematic review, Trial sequential analysis

## Abstract

**Background:**

Theoretically, atrial natriuretic peptide (ANP), especially low-dose ANP, is beneficial in acute kidney injury (AKI). In this study, we examined whether low-dose ANP is effective in preventing or treating AKI by conducting an updated systematic review for randomized controlled trials (RCTs).

**Method:**

We searched the Excerpta Medica database (EMBASE), PubMed, and Cochrane CENTRAL databases for RCTs that compare the effects of low-dose ANP (≤ 50 ng/kg/min) with a placebo or conventional therapy in at-risk patients or patients with AKI. The primary outcome was the incidence of new AKI (in prevention RCTs), while the secondary outcomes were in-hospital mortality rate, renal replacement therapy (RRT) requirement, length of hospital and intensive care unit (ICU) stay, incidence of hypotension, and peak serum creatinine levels. The risk-of-bias was evaluated using the Cochrane Collaboration risk-of-bias tool. Trial sequential analysis (TSA) was used for each outcome of interest.

**Results:**

A total of 18 RCTs (16 prevention and two treatment trials) fulfilled our inclusion criteria. In prevention RCTs, the incidence of new AKI was significantly low in the low-dose ANP group (relative risk [RR] = 0.51; 95% confidence interval [CI] = 0.36–0.72; *P* = 0.0001) compared to the control group. In addition, the low-dose ANP group showed a significantly reduced RRT requirement in both prevention (RR = 0.17; 95% CI = 0.04–0.64; *P* = 0.009) and treatment (RR = 0.43; 95% CI = 0.20–0.93; *P* = 0.03) RCTs. Among secondary outcomes, in some cases, low-dose ANP was associated with a reduction in ICU and in-hospital stay. The risk-of-bias assessment and TSA results indicated that the sample sizes and qualities of the RCTs were insufficient to conclude the efficacy of low-dose ANP.

**Conclusion:**

Low-dose ANP might be effective in preventing or treating AKI. However, the evidence accumulated so far is not strong enough to demonstrate ANP’s beneficial effects. The next step is to elucidate the effects of low-dose ANP by conducting multicenter, high-quality, large-sample RCTs.

**Trial registration:**

PROSPERO registry CRD42017068568. Registered 20 June 2017.

**Electronic supplementary material:**

The online version of this article (10.1186/s13054-019-2330-z) contains supplementary material, which is available to authorized users.

## Background

Acute kidney injury (AKI), a common complication in critically ill patients, is associated with unfavorable clinical outcomes [1]. To improve prognosis in AKI patients, an effective intervention strategy against AKI has to be developed. However, several studies have indicated that, so far, there are no established means to fight AKI [2, 3].

Natriuretic peptides (e.g., atrial natriuretic peptide [ANP] and brain natriuretic peptide [BNP]) are endogenous hormones that are released from the heart in response to myocardial stretch [4]. Through guanylyl cyclase A receptor activation, natriuretic peptides induce pleiotropic actions, such as natriuresis, vasodilation, and suppression of circulating renin, angiotensin II, and aldosterone [4, 5]. ANP also elicits other renal actions, such as an increase in the glomerular filtration rate and the protection of glomerular podocytes from damage [6–8]. Currently, in some countries, human recombinant ANP (carperitide) is approved for acute heart failure (AHF) treatment [9].

Furthermore, natriuretic peptides can be beneficial not only for AHF but also for AKI [10]. Low-dose ANP, especially, may exhibit favorable renal effects without inducing hypotension as indicated by previous studies [11–14]. Therefore, we considered that, in arguing the effect of ANP, the aspect of low-dose infusion should be highlighted and that, theoretically, it has a potential to work as a renoprotective drug. However, it remains elusive whether low-dose ANP is effective for the prevention or treatment of AKI.

In this study, we conducted an updated systematic review and meta-analysis of existing randomized controlled trials (RCTs) that compared low-dose ANP with a placebo or conventional therapy for the prevention or treatment of AKI.

## Methods

### Study protocol

In this study, low dose of ANP was defined as ≤ 50 ng/kg/min in accordance with the AKI guidelines prescribed by the Kidney Disease Improving Global Outcomes (KDIGO) of 2012 [3]. Refer to Additional file 1: Table S1 for a structured outline of our study design using the population, intervention, comparator, and outcome methodology. The phrase “at-risk of AKI” was also defined according to KDIGO AKI guidelines 2012 (Chapter 2.2, “Risk Assessment”) [3]. Our study protocol was in accordance with the Preferred Reporting Items for Systematic Reviews and Meta-Analysis for Protocols 2015 (PRISMA-P 2015) (Additional file 1: Table S2) and A MeaSurement Tool to Assess Systematic Reviews (AMSTAR) guidelines (Additional file 1: Table S3).

The protocol of this study was first registered in the PROSPERO database on July 20, 2017 (no. CRD42017068568) [15]. Since the risk of bias of the included studies and the event rates of some outcomes were different from our expectation, we had to revise the protocol on an as-needed basis. Full details of the revised points can be found on https://www.crd.york.ac.uk/prospero/display_record.php?RecordID=68568.

### Search strategy

A search of the US National Library of Medicine, MEDLINE electronic reference database (PubMed), the Excerpta Medica database (EMBASE), and the Cochrane CENTRAL databases was performed from inception through October 2017. Two investigators (HY and KM) also independently performed a literature search. The search terms used in each electronic database are described in Additional file 1: Table S4. In addition, the references of the identified RCTs and systematic reviews were searched in order to identify further relevant papers.

### Study selection

Two investigators (HY and KM) independently examined the abstracts and titles of the studies identified by literature search in order to exclude irrelevant studies. Inclusion criteria were as follows: (i) RCTs (blinded or unblinded) published between 1970 and October 2017 and (ii) trials comparing low-dose ANP versus a placebo or conventional therapy for prevention or treatment of AKI. The exclusion criteria were as follows: (i) nonhuman experimental studies, (ii) administration of ularitide (a synthetic form of urodilatin excreted by the kidneys) or nesiritide (a trade name of BNP), (iii) administration of high-dose ANP (> 50 ng/kg/min), and (iv) a lack of sufficient data to perform a meta-analysis of targeted outcomes. After selection, the extracted RCTs were divided into prevention and treatment studies. Any discrepancy between the two investigators was independently assessed by a third investigator (KD) and resolved through a consensus among all three investigators.

### Outcomes

The primary outcome of prevention RCTs in this systematic review was the incidence of new AKI, as defined in each trial. The secondary outcomes were as follows: in-hospital mortality rate, renal replacement therapy (RRT) requirement (whose criteria were not necessarily predefined in the RCTs), length of intensive care unit (ICU) stay, length of hospital stay, incidence of hypotension (defined separately in respective RCTs), and peak serum creatinine levels. The outcomes of interest in treatment RCTs were the same as the secondary outcomes of prevention RCTs.

### Data extraction

Prespecified patient and outcome data were independently extracted by two investigators (HY and KM), and any differences between the two were assessed by the third investigator (KD). If the outcome data were incomplete, the original authors of those specific RCTs were contacted by e-mail to obtain the missing information from them.

### Risk-of-bias assessment

Risk-of-bias assessment was performed by two investigators (HY and KM), and disagreements, if any, were resolved by discussion. The Cochrane Collaboration risk-of-bias tool was used to assess the internal validity of the selected RCTs [16, 17].

### Statistical analyses

A pooled analysis was performed using risk ratio (RR) for dichotomous outcomes and the mean difference (MD) for continuous outcomes, with a corresponding 95% confidence interval (CI). The random-effects model was used to pool the results of the RCTs. If the total event rate was ≤ 1%, Peto’s odds ratio method was applied. In addition, to statistically evaluate the effects of confounding factors for the primary outcome, we performed meta-regression analysis; the variables evaluated were age, ANP administration time (> 24 h or < 24 h), use of cardiopulmonary bypass (CPB), and contrast medium. Statistical heterogeneity was assessed by the chi-square test and the *I*^2^ statistic. *P* < 0.10 or *I*^2^ > 50% was an indication of substantial heterogeneity. In the case of considerable heterogeneity (*I*^2^ > 50% or *P <* 0.10), we performed a sensitivity analysis to detect the influence of a single study on the overall estimate by omitting one study in turn and pooling the remaining ones.

In other statistical analyses, *P* < 0.05 was considered statistically significant. Any potential publication bias was assessed by visual assessment of the funnel plots constructed. Meta-regression analysis was performed using Comprehensive Meta-Analysis version 3.0 (Biostat Inc., Englewood, CO, USA), and other statistical analyses were performed using Revman 5.3 (Cochrane IMS, Oxford, UK).

### Trial sequential analysis

Meta-analysis may result in type I errors due to (i) an increased risk of random errors when the data collected are insufficient and (ii) repeated significance testing when a cumulative meta-analysis is updated with new RCTs [18, 19]. To examine the effects of type I errors, we performed trial sequential analysis (TSA) for each outcome [18, 20]. TSA combines the information of size calculation (cumulated sample sizes of all included RCTs) for meta-analysis with a threshold of statistical significance [20]. This threshold adjusts the CIs and reduces type I errors. If the cumulative *Z*-curve crosses the threshold boundaries, the evidence obtained is sufficient to prove ANP’s beneficial effects and no further RCTs are required. In contrast, if the *Z*-curve does not cross any boundary, however, the evidence is insufficient to reach a conclusion [18].

In this study, we conducted TSA with the aim to maintain an overall 5% risk of a type I error and a 20% risk of a type II error, at a power of 80%. To evidence a clinically meaningful difference, we derived the relative risk reduction (RRR) for each outcome from the literature. If the Sidik–Jonkman (S-J) and DerSimonian–Laird (D-L) random-effects models produced different results, a meta-analysis with the two models was conducted and the implications of each scenario were considered true. TSA was conducted with the use of TSA version 0.9 beta (http://www.ctu.dk/tsa) [21].

### Subgroup analysis

Predefined subgroup analysis was conducted on the basis of (i) the clinical setting, cardiovascular surgery and contrast medium; (ii) the control intervention, placebo versus conventional therapy; (iii) the infusion duration, > 24 h and < 24 h; and (iv) removal of RCTs from a single, influential group.

### The GRADE approach

The Grading of Recommendations Assessment, Development, and Evaluation (GRADE) approach was applied to provide an overall assessment of the evidence relating to all of the outcomes. A summary of findings was developed using the GRADEpro software (ims.cochrane.org/revman/other-resources/gradepro) [22].

## Results

### Search results

**Fig. 1:**
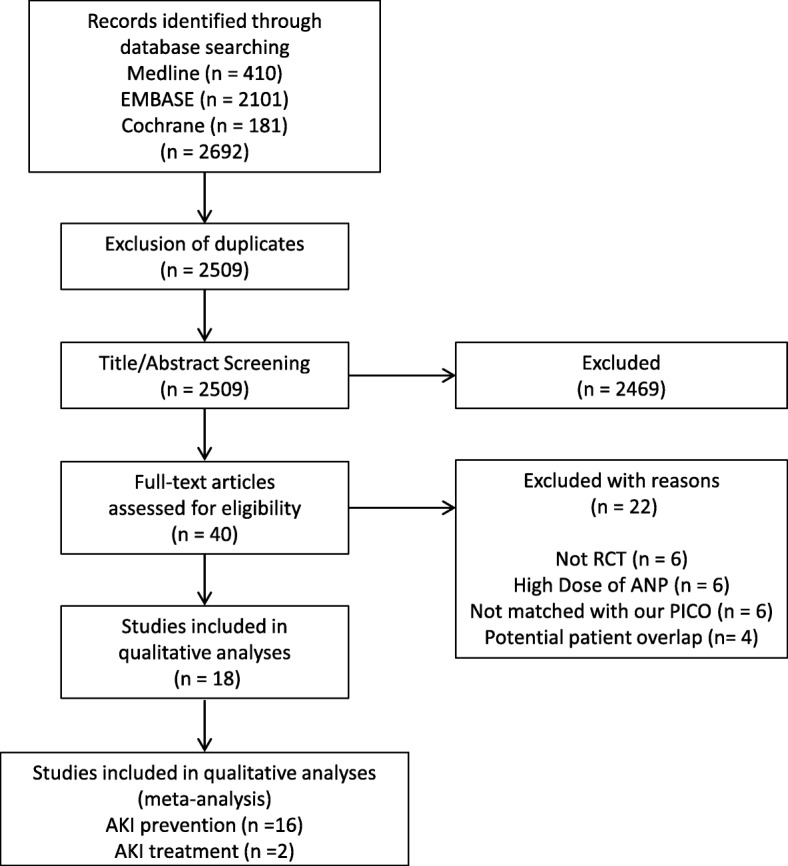
Flowchart of the systematic review and meta-analysis in this study

A flowchart of our search strategy and the reasons for RCT exclusion are shown in Fig. 1. After identification and screening, 40 full-text studies were read for further evaluation and, of these, 18 were excluded because they did not report predefined outcomes or meet our inclusion criteria [13, 14, 23–38]. Additionally, in order to avoid potential overlap of the study population, we excluded some of the studies by Sezai et al. which had been included in previous meta-analysis [39–42] (Additional file 1: Table S5). Finally, the remaining 18 RCTs (16 AKI prevention RCTs and 2 AKI treatment RCTs) were included in the analysis [43–60]. Table 1 lists the baseline characteristics of the included RCTs.

**Table 1:** Baseline characteristics of included studies

Trials	Patients (ANP/control)	Clinical setting (Exposure to AKI risk factors)	Purpose of administration	ANP infusion rate	ANP infusion duration	Comparator (Placebo/Control)	Outcomes
Kurnik BR et al. 1998 [43]	127/60	contrast induced nephropathy	Prevention	0.010 or 0.050 μg/kg/min	< 3 hrs	Placebo (5% dextrose)	Primary: maximum absolute increase in serum creatinine, maximum percent increase in serum creatinine and incidence of CIAKI Secondary: stratification on three concomitant variables: baseline serum creatinine, diabetic status risk group Others: null
Hayashida N et al. 2000 [44]	9/9	mitral valve surgery	Prevention	0.050 μg/kg/min	6 hrs	Control	Primary: not clarified Secondary: not clarified Others: hospital mortality, length of ICU stay, occurrence of hypotension
Hayashi Y et al. 2003 [45]	24/26	aneurysmectomy for abdminal aorta aneurysm	Prevention	0.025 μg/kg/min	> 24 hrs	Control	Primary: not clarified Secondary: not clarified Others: hospital mortality, renal replacement therapy, occurrence of hypotension.
Sward K et al. 2004 [46]	29/30	cardiac surgery	Treatment	0.050 μg/kg/min	> 24 hrs	Placebo (saline)	Primary: dialysis on or before day 21 Secondary: dialysis or death on or before day 21, creatinine clearance on days 1, 2, and 3, length of ICU stay, and ICU mortality Others: null
Sumi K et al. 2008 [47]	30/15	Abdominal aortic aneurysmectomy	Prevention	0.020 or 0.050 μg/kg/min	3 hrs	Placebo (saline)	Primary: not clarified Secondary: not clarified Others: occurrence of hypotension.
Izumi K et al. 2008 [48]	10/8	cardiac surgery	Prevention	0.020 or 0.050 μg/kg/min	> 24hrs	Control	Primary: not clarified Secondary: not clarified Others: hospital mortality, renal replacement therapy, length of ICU stay, length of hospital stay, occurrence of hypotension
Mitaka C et al. 2008 [49]	20/20	abdominal aorta aneurysm repair	Prevention	0.010-0.050 μg/kg/min	> 24hrs	Placebo	Primary: not clarified Secondary: not clarified Others: renal replacement therapy, peak serum creatinine
Hata N et al. 2008 [50]	26/23	acute decompensated heart failure	Prevention	0.010-0.050 μg/kg/min	> 24hrs	Control	Primary: not clarified Secondary: not clarified Others: occurrence of hypotension.
Morikawa S et al. 2009 [51]	126/128	contrast induced nephropathy	Prevention	0.042 μg/kg/min	> 24hrs	Placebo (Ringer solution)	Primary: a 25% increase in creatinine or an increase in creatinine of >0.5 mg/dl from baseline within 48 hr Secondary: 1) a 25% increase in creatinine within 48 hr; 2) an increase in creatinine of >0.5 mg/dl from baseline within 48 hr; 3) changes in serum creatinine, eGFR and serum cystatin C concentrations, and urinary β2-microglobulin and NAG until 1 month after the procedure; and 4) a 25% increase in creatinine or an increase in creatinine of > 0.5 mg/dl from baseline at 1 month after the procedure. Others: renal replacement therapy
Sezai A et al. 2009 [52]	251/253	CABG	Prevention	0.010-0.020 μg/kg/min	> 24hrs	Placebo (saline)	Primary: not clarified Secondary: not clarified Others: hospital mortality, renal replacement therapy, length of hospital stay, acute kidney injury (0.3mg/dl > pre-operative - maxium Cr), occurrence of hypotension, peak serum creatinine
Sezai A et al. 2011 [53]	141/144	CABG	Prevention	0.010-0.020 μg/kg/min	> 24hrs	Placebo	Primary:1) dialysis-free rate at 1 year post-operatively, 2) sCr and eGFR at 0, 1, and 3 days, 1 week, and 1 month post-operatively Secondary: 1) the early post-operative outcome (operative mortality and complications), 2) outcome at 1 year post-operatively (overall survival rate and cardiac event-free rate), 3) the maximum sCr, the rate of increase of sCr (% Cr: [maximum sCr pre-operative sCr]/pre-operative sCr × 100), and an increase of sCr by 0.3 mg/dl compared with the pre-operative value, 4) ANP and cyclic-guanosine monophosphate levels (on return to intensive care unit, and on postoperative day 1, week 1, and month 1) Others: renal replacement therapy, length of ICU stay, occurrence of hypotension, peak serum creatinine
Tamura Y et al. 2011 [54]	19/20	liver resection	Prevention	0.025 μg/kg/min	6 hrs	Control	Primary: not clarified Secondary: not clarified Others: length of ICU stay, length of hospital stay, acute kidney injury (0.3mg/dl > pre-operative - maxium Cr), peak serum creatinine
Okumura N et al. 2012 [55]	59/53	contrast-induced nephropathy	Prevention	0.013-0.025 μg/kg/min	18-24 hrs	Placebo (Saline)	Primary: the occurrence of CIAKI Secondary: theserum creatinine and cystatin C levels Others: occurrence of hypotension
Hisatomi K et al. 2012 [56]	40/30	cardiovascular surgery	Prevention	0.010-0.020 μg/kg/min	> 24 hrs	Control	Primary: serum Cr level 3 days after surgery Secondary: serum Cr levels at each time point of measurement within 3 days after surgery Others: hospital mortality, renal replacement therapy, occurrence of hypotension.
Wang P et al. 2013 [57]	12/12	acute decompensated heart failure	Prevention	0.050 μg/kg/min	1 hr	Control	Primary: absolute changes in PCWP from baseline to 1 hr after the start of study drug Secondary: the effect on PCWP 0.5, 3 and 12 hr after the start of study drug, the effect on CO and SV at 0.5, 1, 3 and 12 hr, patient’s self-evaluation of dyspnoea, urine volume and the overall safety profile Others: hospital mortality, occurrence of hypotension
Mori Y et al. 2014 [58]	20/22	aortic arch aneurysm repair	Prevention	0.0125 μg/kg/min	> 24 hrs	Placebo (5% glucose)	Primary: occurrence of AKI within 48hr of surgery Secondary: occurrence of dialysis and/or all-cause mortality in the first 30 postoperative days Others: length of ICU stay, length of hospital stay, occurrence of hypotension and atrial fibrillation.
Moriyama T et al. 2017 [59]	24/24	cardiac surgery	Prevention	0.025 μg/kg/min,	> 24 hrs	Placebo (5% Salie)	Primary: the occurrence of AKI Secondary: not clarified Others: occurrence of hypotension.
Mitaka C et al. 2017 [60]	37/40	cardiovascular surgery	Treatment	0.020 μg/kg/min,	> 24hrs	Placebo(5% glucose)	Primary: change in renal function over the 90-day follow up Secondary: 1) a need for renal replacement therapy over the 90-day follow-up, 2) the lengths of ICU and hospital stays, 3) medical costs incurred over the 90-day follow-up Others: occurrence of hypotension.

*Abbreviations*: *AKI* acute kidney injury, *ANP* atrial natriuretic peptide, *BNP* brain natriuretic peptide, *CABG* coronary artery bypass grafting, *CIAKI* contrast-induced acute kidney injury, *CO* cardiac output, *CPK* creatine kinase, *Cr* creatinine, *EF* ejection fraction, *eGFR* estimated glomerular filtration rate, *ICU* intensive care unit, *NAG* N-Acetyl Glucosaminidase, *PCWP* pulmonary capillary wedge pressure, *sCr* serum creatinine, *SV* stroke volume

### Risk of bias in the included RCTs

**Fig. 2:**
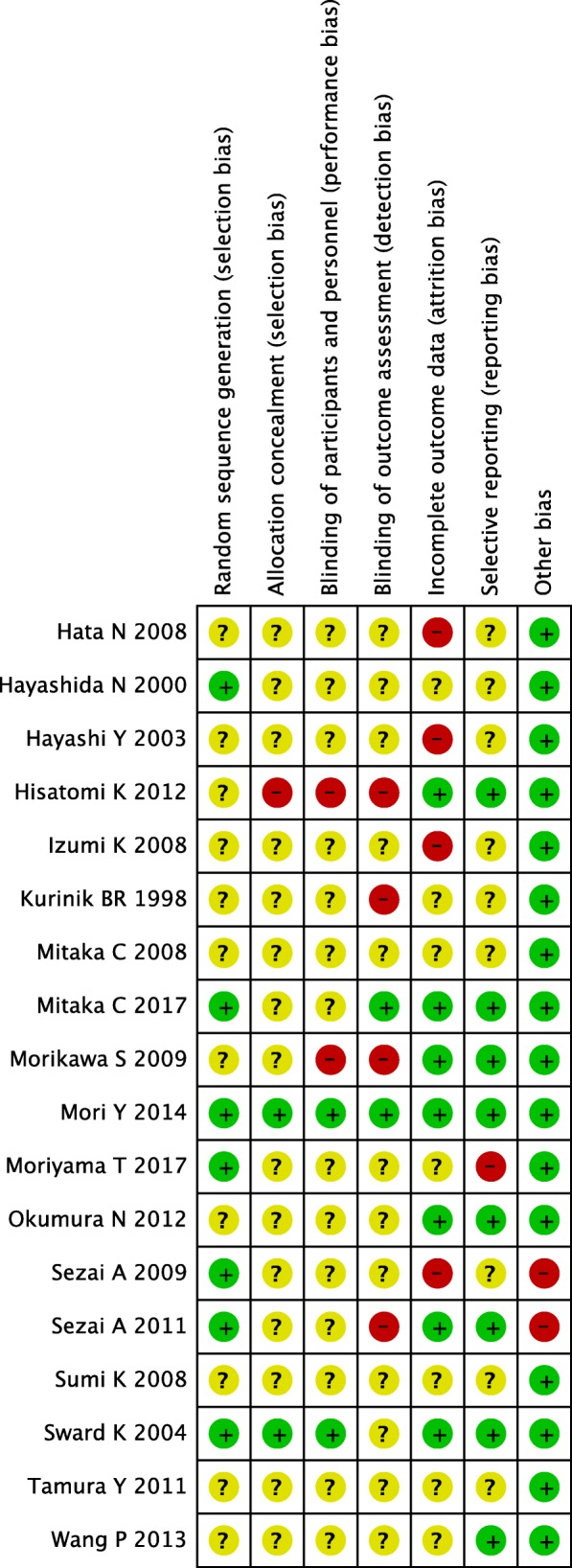
Risk-of-bias assessment. A review of investigators’ judgment about each risk-of-bias domain for each included RCT is shown. Red circles indicate high risk, green circles indicate low risk, and yellow circles indicate unclear risk. RCT, randomized controlled trial

Our risk-of-bias assessment for each of the 18 RCTs is shown in Fig. 2. Most of them presented a high or an unclear risk of bias in three domains: allocation concealment, blinding of participants and personnel, and blinding outcome assessment. Only one RCT presented a low risk of bias in all three domains [58]. Twelve RCTs reported funding sources or declared no conflicts of interest [43, 44, 46, 47, 49, 50, 52, 53, 55, 58–60], and six RCTs did not specify their funding sources [45, 48, 51, 54, 56, 57]. No RCT reported funding from the industry.

### ANP for AKI prevention

#### Incidence of AKI

**Fig. 3:**
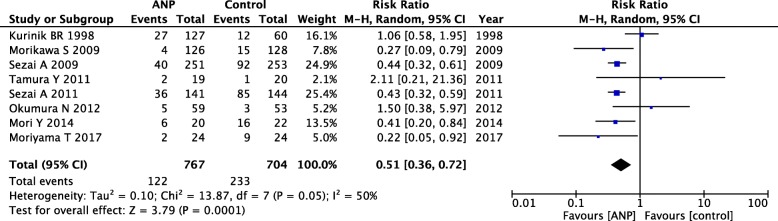
Forest plot of AKI incidence in prevention RCTs. CI, confidential interval; M-H, Mantel–Haenszel; AKI, acute kidney injury; RCT, randomized controlled trial

In this study, eight prevention RCTs reported the incidence of new AKI. The pooled estimate was significantly low in the low-dose ANP group (relative risk [RR] = 0.51; 95% CI = 0.36–0.72; *P* = 0.0001), compared to the control group, although with mild heterogeneity (*P* = 0.05; *I*^2^ = 50%; Fig. 3). Therefore, we performed a sensitivity analysis to detect the source of heterogeneity. When we omitted Kurnik’s trial [43], the heterogeneity reached an acceptable level and the significance of the pooled estimate remained stable (Additional file 1: Table S6).

Meta-regression analysis showed that ANP administration time of > 24 h strengthened the positive effect of low-dose ANP by significantly decreasing AKI incidence (*P* = 0.047) (Additional file 1: Figure S1). On the other hand, we did not find a significant correlation between AKI incidence and other confounding factors, including age (*P* = 0.96), the use of CPB (*P* = 0.90), or contrast medium (*P* = 0.60).

In TSA, the D-L random-effects model findings showed that although the sample size did not reach the required information size, the cumulative *Z*-curve crossed both the conventional and trial sequential monitoring boundaries for benefits (Additional file 1: Figure S2). In contrast, the S-J random-effects model results showed that because of the heterogeneity of the included RCTs, the cumulative *Z*-curve only partially crossed the conventional boundary for benefits (Additional file 1: Figure S3). These results indicated the necessity for future multicenter, high-quality, large-sample RCTs.

#### In-hospital mortality

**Fig. 4:**
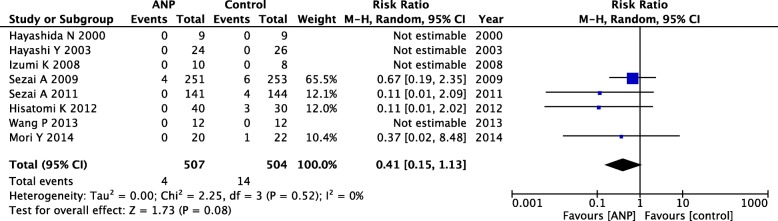
Forest plot of in-hospital mortality rate in prevention RCTs. ANP, atrial natriuretic peptide; CI, confidential interval; M-H, Mantel–Haenszel; RCT, randomized controlled trial

In-hospital mortality rates were reported in eight prevention RCTs. A forest plot showed that low-dose ANP did not have a beneficial effect on the in-hospital mortality rate (RR = 0.41; 95% CI = 0.15–1.13; *P* = 0.08), with no apparent heterogeneity (*P* = 0.52; *I*^2^ = 0%; Fig. 4). In TSA, the cumulative *Z*-curve did not cross any of the threshold boundaries, and we did not observe any significant effects on the in-hospital mortality rates (Additional file 1: Figure S4).

### Need for renal replacement therapy

**Fig. 5:**
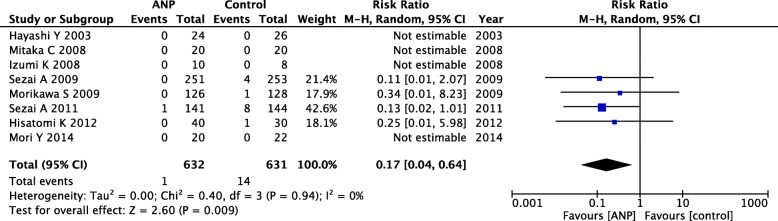
Forest plot of RRT in prevention RCTs. ANP, atrial natriuretic peptide; CI, confidential interval; M-H, Mantel–Haenszel; RRT, renal replacement therapy

Data on RRT requirement were available in eight prevention RCTs. While 2.2% of the patients in the control group received RRT during follow-up, this figure was only 0.2% in the low-dose ANP group. A forest plot of RRT showed a significant difference between the two groups (RR = 0.17; 95% CI = 0.04–0.64; *P* = 0.009), with no apparent heterogeneity (*P* = 0.94; *I*^2^ = 0%; Fig. 5). In TSA, the cumulative *Z*-curve crossed only the conventional boundary for benefits and the sample size did not reach the required information size (Additional file 1: Figure S5).

#### Hospital and ICU stay

The length of hospital stay was reported in six prevention RCTs. It was significantly shorter in the low-dose ANP group (MD = − 2.65 days; 95% CI = − 4.45 to − 0.86; *P* = 0.004), compared to the control group, with no apparent heterogeneity (*P* = 0.16; *I*^2^ = 38%; Additional file 1: Figure S6). The length of ICU stay was reported in six preventive RCTs, with no significant difference between the two groups (MD = 0.06 days; 95% CI = − 0.31 to 0.43; *P* = 0.75) and with no apparent heterogeneity (*P* = 0.27; *I*^2^ = 23%; Additional file 1: Figure S6).

In TSA for the hospital stay period, the cumulative *Z*-curve crossed only the conventional boundary for benefits and the sample size did not reach the required information size (Additional file 1: Figure S7). We could not apply TSA to comparisons of the length of ICU stay because the sample size was too small.

#### Incidence of hypotension

Since the total event rate in the incidence of hypotension was < 1%, we applied Peto’s odds ratio method. Forest plots indicated that low-dose ANP significantly induces hypotension (odds ratio = 8.57; 95% CI = 3.88–18.95; *P* < 0.001), with significant heterogeneity (*P* = 0.03; *I*^2^ = 78%; Additional file 1: Figure S8). It was impossible to perform sensitivity analysis and apply TSA to the comparisons because the number of RCTs and the sample size were too small.

#### Peak serum creatinine levels

Forest plots showed that low-dose ANP did not significantly reduce the peak serum creatinine levels (MD = − 0.18 mg/dL; 95% CI = − 0.38 to 0.01; *P* = 0.07), with significant heterogeneity (*P* = 0.007; *I*^2^ = 75%; Additional file 1: Figure S9). In a sensitivity analysis, whichever study was omitted, the heterogeneity remained significant (Additional file 1: Table S7). We could not apply TSA to this outcome because the sample size was too small.

#### Subgroup analysis

Additional file 1: Table S8 shows the summary of our subgroup analysis. Although the results were not radically different from the overall results, the original significance was not evident in some comparisons. In particular, when the two high-volume RCTs from Sezai’s group were removed from the analysis, significant reduction in the rate of new AKI by low-dose ANP was not detected (RR = 0.60; 95% CI = 0.31–1.15; *P* = 0.05) [52, 53].

### ANP for AKI treatment

**Fig. 6:**
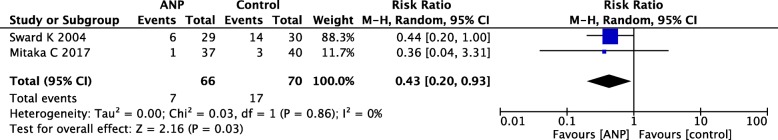
Forest plot of RRT in treatment RCTs. ANP, atrial natriuretic peptide; CI, confidential interval; M-H, Mantel–Haenszel; RRT, renal replacement therapy

In this study, only two treatment RCTs met our inclusion criteria. Compared to the control group, the low-dose ANP group showed a significantly reduced need for RRT (RR = 0.43; 95% CI = 0.20–0.93; *P* = 0.03; Fig. 6) and shortened length of ICU stay (MD = − 2.41 days; 95% CI = − 3.49 to − 1.34; *P* < 0.0001; Additional file 1: Figure S10). In addition, the incidence of hypotension was not significantly more frequent in the low-dose ANP group (RR = 1.27; 95% CI = 0.64–2.50; *P* = 0.50) (Additional file 1: Figure S11) compared to the control group. With regard to other outcomes, it was impossible to calculate summary statistics because they were not reported in both treatment RCTs. Moreover, we could not perform subgroup analysis because of the low number of included RCTs.

In TSA of RRT requirement, the cumulative *Z*-curve crossed only the conventional boundary for benefits (Additional file 1: Figure S12). However, in TSA of the length of ICU stay, the cumulative *Z*-curve crossed both the conventional and trial sequential monitoring boundaries for benefits and reached the required information size (Additional file 1: Figure S13). The results indicated sufficient and conclusive evidence to prove ANP’s beneficial effects and showed that further RCTs are not required. Meanwhile, the cumulative *Z*-curve of the incidence of hypotension did not cross any of the threshold boundaries (Additional file 1: Figure S14).

### Funnel plots

To determine publication bias, we created funnel plots (Additional file 1: Figure S15 and S16). The shapes of the funnel plots did not show obvious asymmetry in each outcome’s analysis.

### GRADEpro summary of findings

Additional file 1: Table S9 and S10 show our GRADE evidence profile. The quality of evidence was assessed as low or very low in most of the outcomes because of high risk of bias and limited sample size.

## Discussion

In this study, we investigated the effect of low-dose ANP on AKI prevention or treatment. Low-dose ANP significantly reduced the incidence of new AKI and RRT requirement for both at-risk patients and patients with AKI. In addition, low-dose ANP shortened the hospital or ICU stay, depending on the situation. However, our findings were not conclusive, because the quality of evidence for each outcome was quite low. Indeed, we had to partially revise the protocol due to high risk of bias in most of the included studies. In addition, the sample size was not large enough to demonstrate significant effects, except for the length of ICU stay in treatment RCTs.

Previous studies conducted in 2009 indicated the benefit of ANP for AKI [11, 12]. The studies also showed a trend toward RRT reduction and a good safety profile in the low-dose ANP group [11, 12]. After the last study was published, many RCTs were conducted to investigate the protective effects of low-dose ANP [42, 48–53, 56, 58–60]. Unfortunately, however, our study, including recent RCTs, could not firmly demonstrate a positive effect of low-dose ANP on AKI prevention or treatment. In fact, in most of the outcomes, TSA showed insufficiency of the sample size. Therefore, this study re-emphasized the sheer necessity of multicenter, high-quality, large-sample RCTs.

One of the strengths of this study was the revelation of the importance of performing treatment RCTs with low-dose ANP. Previous systematic reviews including high-dose ANP studies have reported that the incidence of hypotension is significantly higher in the ANP group [11, 12]. However, our inclusion criteria limited the infusion of ANP to a low-dose; therefore, our meta-analysis of treatment RCTs did not show a significant difference in the incidence of hypotension. Nevertheless, our study raised the possibility of reduction in RRT requirement and the length of ICU stay in treatment RCTs.

In our meta-analysis of prevention RCTs, on the other hand, the incidence of hypotension was significantly higher in the low-dose ANP group compared to the control group. Of note, Okumura et al. [55] defined hypotension as the absolute reduction of systematic blood pressure and reported that low-dose ANP significantly increases the incidence of hypotension. In contrast, other studies referred to the apparent episodes associated with ANP administration as hypotension. We believe that this difference in the definition of hypotension mainly influenced the results of this study.

This study had a few limitations. First, the definition of AKI was considerably heterogeneous among the included RCTs. Some recent RCTs defined AKI on the basis of the KDIGO 2012 guidelines, while others defined AKI as a rise in the serum creatinine level by 0.5 mg/dL. Second, we were unable to ignore selection bias. In fact, more than half of the RCTs included in this study were single-center studies [44–49, 51, 53–55, 58, 59]. In addition, a recent retrospective cohort study with a large-sample size from the Japanese Diagnosis Procedure Combination database did not report a positive effect of low-dose ANP on AKI prevention or treatment [61, 62]. Although our study design and data analysis were entirely different from the retrospective study, we need to keep in mind that the difference in the results might be due to selection bias or limited sample size. Third, it might be inappropriate to directly apply these results to ICU patients. Most of the participants in the included studies were cardiovascular surgery patients. Additionally, as most of the included studies in our analysis were AKI prevention trials, the severity of the participants might be relatively low compared to medical ICU patients. Therefore, our results should not be generalized in all of the ICU patients. Fourth, we partially revised the protocol in terms of study selection, outcome, and subgroup analysis after starting the meta-analysis. Therefore, we cannot completely deny the possibility of extraction bias.

## Conclusions

This study indicated that if low-dose ANP is administered to prevent or treat AKI, it can reduce AKI incidence and RRT requirement. In addition, it can shorten the length of ICU or hospital stay in some situations. However, in this study, the quality and sample size of the RCTs included were not sufficient for demonstrating the beneficial effects of low-dose ANP on AKI prevention or treatment. In the future, to elucidate the effects of low-dose ANP, it is necessary to perform multicenter, high-quality, large-sample RCTs.

## Additional file

Additional file 1: **Table S1.** PICO model. **Table S2.** PRISMA-P 2015 Checklist. **Table S3.** AMSTAR Checklist. **Table S4.** Terms used to search the electronic databases. **Table S5.** Excluded studies with full-text reading. **Table S6.** Sensitivity analysis of incidence of acute kidney injury in the prevention trials. **Table S7.** Sensitivity analysis of peak serum creatinine in the prevention trials. **Table S8.** Subgroup analysis. **Table S9.** GRADE pro summary of finding tables for the prevention of AKI. **Table S10.** GRADE pro summary of finding tables for the treatment of AKI. **Figure S1.** Meta-regression results for the reduction of acute kidney injury by low-dose ANP. **Figure S2.** Trial sequential analysis: acute kidney injury in the prevention trials (random-effects D-L). **Figure S3.** Trial sequential analysis of acute kidney injury in the prevention trials (random-effects S-J). **Figure S4.** Trial sequential analysis of acute kidney injury in the prevention trials (random-effects D-L). **Figure S5.** Trial sequential analysis of renal replacement therapy in the prevention trials (random-effects D-L). **Figure S6.** Forest plot for hospital stay and ICU stay in the prevention trials. **Figure S7.** Trial sequential analysis: hospital stay in the prevention trials (random-effects D-L). **Figure S8.** Forest plot for occurrence of hypotension in the prevention trials. **Figure S9.** Forest plot for peak serum creatinine in the prevention trials. **Figure S10.** Forest plot for ICU stay in the treatment trials. **Figure S11.** Forest plot for occurrence of hypotension in the treatment trials. **Figure S12.** Trial sequential analysis of renal replacement therapy in the treatment trials. **Figure S13.** Trial sequential analysis of ICU stay in the treatment trials. **Figure S14.** Trial sequential analysis of occurrence of hypotension in the treatment trials. **Figure S15.** Funnel plots in the prevention trials. **Figure S16.** Funnel plots in the treatment trials. (DOCX 60973 kb)

## Abbreviations

AKI: Acute kidney injury
ANP: Atrial natriuretic peptide
BNP: Brain natriuretic peptide
CI: Confidence interval
D-L: DerSimonian–Laird
ICU: Intensive care unit
KDIGO: Kidney Disease Improving Global Outcomes
MD: Mean difference
RCT: Randomized controlled trial
RR: Risk ratio
RRR: Relative risk reduction
RRT: Renal replacement therapy
S-J: Sidik–Jonkman
TSA: Trial sequential analysis

## References

[1] Murugan R, Kellum JA (2011). Acute kidney injury: what’s the prognosis?. Nat Rev Nephrol.

[2] National Clinical Guideline C: National Institute for Health and Clinical Excellence: Guidance. In: Acute kidney injury: prevention, detection and management up to the point of renal replacement therapy*.* London: Royal College of Physicians (UK) National Clinical Guideline Centre 2013. 25340231

[3] Group KDIGO AKI Working Group (2012). KDIGO clinical practice guideline for acute kidney injury. Kidney Int Suppl.

[4] Potter LR, Abbey-Hosch S, Dickey DM (2006). Natriuretic peptides, their receptors, and cyclic guanosine monophosphate-dependent signaling functions. Endocr Rev.

[5] Kuwahara K, Nakao K (2010). Regulation and significance of atrial and brain natriuretic peptides as cardiac hormones. Endocr J.

[6] Marin-Grez M, Fleming JT, Steinhausen M (1986). Atrial natriuretic peptide causes pre-glomerular vasodilatation and post-glomerular vasoconstriction in rat kidney. Nature.

[7] Ogawa Y, Mukoyama M, Yokoi H, Kasahara M, Mori K, Kato Y, Kuwabara T, Imamaki H, Kawanishi T, Koga K (2012). Natriuretic peptide receptor guanylyl cyclase-A protects podocytes from aldosterone-induced glomerular injury. J Am Soc Nephrol.

[8] Kato Y, Mori K, Kasahara M, Osaki K, Ishii A, Mori KP, Toda N, Ohno S, Kuwabara T, Tokudome T (2017). Natriuretic peptide receptor guanylyl cyclase-A pathway counteracts glomerular injury evoked by aldosterone through p38 mitogen-activated protein kinase inhibition. Sci Rep.

[9] Lee CY, Burnett JC (2007). Natriuretic peptides and therapeutic applications. Heart Fail Rev.

[10] Vesely DL (2003). Natriuretic peptides and acute renal failure. Am J Physiol Renal Physiol.

[11] Nigwekar SU, Navaneethan SD, Parikh CR, Hix JK (2009). Atrial natriuretic peptide for management of acute kidney injury: a systematic review and meta-analysis. Clin J Am Soc Nephrol.

[12] Nigwekar SU, Navaneethan SD, Parikh CR, Hix JK (2009). Atrial natriuretic peptide for preventing and treating acute kidney injury. Cochrane Database Syst Rev.

[13] Lewis J, Salem MM, Chertow GM, Weisberg LS, McGrew F, Marbury TC, Allgren RL (2000). Atrial natriuretic factor in oliguric acute renal failure. Anaritide Acute Renal Failure Study Group. Am J Kidney Dis.

[14] Allgren RL, Marbury TC, Rahman SN, Weisberg LS, Fenves AZ, Lafayette RA, Sweet RM, Genter FC, Kurnik BR, Conger JD (1997). Anaritide in acute tubular necrosis. Auriculin Anaritide Acute Renal Failure Study Group. N Engl J Med.

[15] Shamseer L, Moher D, Clarke M, Ghersi D, Liberati A, Petticrew M, Shekelle P, Stewart LA (2015). Preferred reporting items for systematic review and meta-analysis protocols (PRISMA-P) 2015: elaboration and explanation. BMJ.

[16] Higgins JP, Altman DG, Gotzsche PC, Juni P, Moher D, Oxman AD, Savovic J, Schulz KF, Weeks L, Sterne JA (2011). The Cochrane Collaboration’s tool for assessing risk of bias in randomised trials. BMJ.

[17] Higgins JPT GSe: Cochrane Handbook for Systematic Reviews of Interventions Version 5.1.0 [updated March 2011]. Cochrane Collaboration**,** 2011**.** Available from www.handbook.cochrane.org. Accessed 1 Jan 2019.

[18] Wetterslev J, Thorlund K, Brok J, Gluud C (2008). Trial sequential analysis may establish when firm evidence is reached in cumulative meta-analysis. J Clin Epidemiol.

[19] Brok J, Thorlund K, Wetterslev J, Gluud C (2009). Apparently conclusive meta-analyses may be inconclusive--trial sequential analysis adjustment of random error risk due to repetitive testing of accumulating data in apparently conclusive neonatal meta-analyses. Int J Epidemiol.

[20] Wetterslev J, Thorlund K, Brok J, Gluud C (2009). Estimating required information size by quantifying diversity in random-effects model meta-analyses. BMC Med Res Methodol.

[21] Kristian Thorlund JE, Wetterslev J, Brok J, Imberger G, Gluud C (2011). User manual for trial sequential analysis (TSA).

[22] Holger Schünemann JB, Guyatt G, Oxman A (2013). GRADE handbook for grading the quality of evidence and the strength of recommendations using the GRADE approach.

[23] Woolf AS, Mansell MA, Hoffbrand BI, Cohen SL, Moult PJ (1989). The effects of low dose intravenous 99-126 atrial natriuretic factor infusion in patients with chronic renal failure. Postgrad Med J.

[24] Kurnik BR, Weisberg LS, Cuttler IM, Kurnik PB (1990). Effects of atrial natriuretic peptide versus mannitol on renal blood flow during radiocontrast infusion in chronic renal failure. J Lab Clin Med.

[25] Ratcliffe PJ, Richardson AJ, Kirby JE, Moyses C, Shelton JR, Morris PJ (1991). Effect of intravenous infusion of atriopeptin 3 on immediate renal allograft function. Kidney Int.

[26] Sands JM, Neylan JF, Olson RA, O'Brien DP, Whelchel JD, Mitch WE (1991). Atrial natriuretic factor does not improve the outcome of cadaveric renal transplantation. J Am Soc Nephrol.

[27] Lang CC, Henderson IS, Mactier R, Stewart WK, Struthers AD (1992). Atrial natriuretic factor improves renal function and lowers systolic blood pressure in renal allograft recipients treated with cyclosporin A. J Hypertens.

[28] Rahman SN, Kim GE, Mathew AS, Goldberg CA, Allgren R, Schrier RW, Conger JD (1994). Effects of atrial natriuretic peptide in clinical acute renal failure. Kidney Int.

[29] Bergman A, Odar-Cederlof I, Westman L, Ohqvist G (1996). Effects of human atrial natriuretic peptide in patients after coronary artery bypass surgery. J Cardiothorac Vasc Anesth.

[30] Akamatsu N, Sugawara Y, Tamura S, Kaneko J, Togashi J, Kishi Y, Imamura H, Kokudo N, Makuuchi M (2005). Prevention of renal impairment by continuous infusion of human atrial natriuretic peptide after liver transplantation. Transplantation.

[31] Sezai A, Shiono M, Hata M, Iida M, Wakui S, Soeda M, Negishi N, Kasamaki Y, Saito S, Kato J (2006). Efficacy of continuous low-dose human atrial natriuretic peptide given from the beginning of cardiopulmonary bypass for thoracic aortic surgery. Surg Today.

[32] Yoshitake I, Sezai A, Hata M, Niino T, Unosawa S, Wakui S, Shiono M (2011). Low-dose atrial natriuretic peptide for chronic kidney disease in coronary surgery. Ann Thorac Cardiovasc Surg.

[33] Suzuki S, Yoshihisa A, Yamaki T, Sugimoto K, Kunii H, Nakazato K, Abe Y, Saito T, Ohwada T, Suzuki H (2014). Long-term effects and prognosis in acute heart failure treated with tolvaptan: the AVCMA trial. Biomed Res Int.

[34] Wang G, Wang P, Li Y, Liu W, Bai S, Zhen Y, Li D, Yang P, Chen Y, Hong L (2016). Efficacy and safety of 1-hour infusion of recombinant human atrial natriuretic peptide in patients with acute decompensated heart failure: a phase III, randomized, double-blind, placebo-controlled, multicenter trial. Med.

[35] Takaya Y, Yoshihara F, Yokoyama H, Kanzaki H, Kitakaze M, Goto Y, Anzai T, Yasuda S, Ogawa H, Kawano Y (2017). Impact of decreased serum albumin levels on acute kidney injury in patients with acute decompensated heart failure: a potential association of atrial natriuretic peptide. Heart Vessel.

[36] Tsukamoto M, Koyama S, Esaki K, Hitosugi T, Yokoyama T (2017). Low-dose carperitide (alpha-human A-type natriuretic peptide) alleviates hemoglobin concentration decrease during prolonged oral surgery: a randomized controlled study. J Anesth.

[37] Sezai A, Nakata K, Iida M, Yoshitake I, Wakui S, Hata H, Shiono M (2013). Results of low-dose carperitide infusion in high-risk patients undergoing coronary artery bypass grafting. Ann Thorac Surg.

[38] Sezai A, Nakata K, Iida M, Yoshitake I, Wakui S, Hata H, Shiono M (2014). Early results of human atrial natriuretic peptide infusion in non-dialysis patients with chronic kidney disease undergoing isolated coronary artery bypass grafting: the NU-HIT trial for CKD-II. Ann Thorac Cardiovasc Surg.

[39] Sezai A, Shiono M, Orime Y, Hata H, Hata M, Negishi N, Sezai Y (2000). Low-dose continuous infusion of human atrial natriuretic peptide during and after cardiac surgery. Ann Thorac Surg.

[40] Sezai A, Hata M, Wakui S, Shiono M, Negishi N, Kasamaki Y, Saito S, Kato J, Minami K (2006). Efficacy of low-dose continuous infusion of alpha-human atrial natriuretic peptide (hANP) during cardiac surgery: possibility of postoperative left ventricular remodeling effect. Circ J.

[41] Sezai A, Hata M, Wakui S, Niino T, Takayama T, Hirayama A, Saito S, Minami K (2007). Efficacy of continuous low-dose hANP administration in patients undergoing emergent coronary artery bypass grafting for acute coronary syndrome. Circ J.

[42] Sezai A, Hata M, Niino T, Yoshitake I, Unosawa S, Wakui S, Fujita K, Takayama T, Kasamaki Y, Hirayama A (2010). Continuous low-dose infusion of human atrial natriuretic peptide in patients with left ventricular dysfunction undergoing coronary artery bypass grafting: the NU-HIT (Nihon University working group study of low-dose human ANP infusion therapy during cardiac surgery) for left ventricular dysfunction. J Am Coll Cardiol.

[43] Kurnik BR, Allgren RL, Genter FC, Solomon RJ, Bates ER, Weisberg LS (1998). Prospective study of atrial natriuretic peptide for the prevention of radiocontrast-induced nephropathy. Am J Kidney Dis.

[44] Hayashida N, Chihara S, Kashikie H, Tayama E, Yokose S, Akasu K, Aoyagi S (2000). Effects of intraoperative administration of atrial natriuretic peptide. Ann Thorac Surg.

[45] Hayashi Y, Ohtani M, Sawa Y, Hiraishi T, Akedo H, Kobayashi Y, Matsuda H (2003). Synthetic human alpha-atrial natriuretic peptide improves the management of postoperative hypertension and renal dysfunction after the repair of abdominal aortic aneurysm. J Cardiovasc Pharmacol.

[46] Sward K, Valsson F, Odencrants P, Samuelsson O, Ricksten SE (2004). Recombinant human atrial natriuretic peptide in ischemic acute renal failure: a randomized placebo-controlled trial. Crit Care Med.

[47] Sumi K, Iida H, Yamaguchi S, Fukuoka N, Shimabukuro K, Dohi S (2008). Human atrial natriuretic peptide prevents the increase in pulmonary artery pressure associated with aortic unclamping during abdominal aortic aneurysmectomy. J Cardiothorac Vasc Anesth.

[48] Izumi K, Eishi K, Yamachika S, Hashizume K, Tada S, Yamane K, Takai H, Tanigawa K, Miura T, Nakaji S (2008). The efficacy of human atrial natriuretic peptide in patients with renal dysfunction undergoing cardiac surgery. Ann Thorac Cardiovasc Surg.

[49] Mitaka C, Kudo T, Jibiki M, Sugano N, Inoue Y, Makita K, Imai T (2008). Effects of human atrial natriuretic peptide on renal function in patients undergoing abdominal aortic aneurysm repair. Crit Care Med.

[50] Hata N, Seino Y, Tsutamoto T, Hiramitsu S, Kaneko N, Yoshikawa T, Yokoyama H, Tanaka K, Mizuno K, Nejima J (2008). Effects of carperitide on the long-term prognosis of patients with acute decompensated chronic heart failure: the PROTECT multicenter randomized controlled study. Circ J.

[51] Morikawa S, Sone T, Tsuboi H, Mukawa H, Morishima I, Uesugi M, Morita Y, Numaguchi Y, Okumura K, Murohara T (2009). Renal protective effects and the prevention of contrast-induced nephropathy by atrial natriuretic peptide. J Am Coll Cardiol.

[52] Sezai A, Hata M, Niino T, Yoshitake I, Unosawa S, Wakui S, Osaka S, Takayama T, Kasamaki Y, Hirayama A (2009). Influence of continuous infusion of low-dose human atrial natriuretic peptide on renal function during cardiac surgery: a randomized controlled study. J Am Coll Cardiol.

[53] Sezai A, Hata M, Niino T, Yoshitake I, Unosawa S, Wakui S, Kimura H, Shiono M, Takayama T, Hirayama A (2011). Results of low-dose human atrial natriuretic peptide infusion in nondialysis patients with chronic kidney disease undergoing coronary artery bypass grafting: the NU-HIT (Nihon University working group study of low-dose HANP infusion therapy during cardiac surgery) trial for CKD. J Am Coll Cardiol.

[54] Tamura Y, Nagata H, Sato Y, Nitta H, Wakabayashi G (2011). Usefulness of human atrial natriuretic peptide (hANP) on perioperative management for liver resection. Masui Japanese J Anesthesiol.

[55] Okumura N, Hayashi M, Imai E, Ishii H, Yoshikawa D, Yasuda Y, Goto M, Matsuo S, Oiso Y, Murohara T (2012). Effects of carperitide on contrast-induced acute kidney injury with a minimum volume of contrast in chronic kidney disease patients. Nephron Extra.

[56] Hisatomi K, Eishi K (2012). Multicenter trial of carperitide in patients with renal dysfunction undergoing cardiovascular surgery. Gen Thorac Cardiovasc Surg.

[57] Wang P, Luan X, Wang G, Liu W, Zhang J, Li W, Gao X, Wang Y, Mao Y, Sun X (2013). Efficacy and safety of short-term administration of recombinant human atrial natriuretic peptide (rhANP) for congestive heart failure: a phase II, multicentre randomized controlled dose-finding study. J Clin Pharm Ther.

[58] Mori Y, Kamada T, Ochiai R (2014). Reduction in the incidence of acute kidney injury after aortic arch surgery with low-dose atrial natriuretic peptide: a randomised controlled trial. Eur J Anaesthesiol.

[59] Moriyama T, Hagihara S, Shiramomo T, Nagaoka M, Iwakawa S, Kanmura Y (2017). The protective effect of human atrial natriuretic peptide on renal damage during cardiac surgery. J Anesth.

[60] Mitaka C, Ohnuma T, Murayama T, Kunimoto F, Nagashima M, Takei T, Iguchi N, Tomita M (2017). Effects of low-dose atrial natriuretic peptide infusion on cardiac surgery-associated acute kidney injury: a multicenter randomized controlled trial. J Crit Care.

[61] Sasabuchi Y, Yasunaga H, Matsui H, Lefor AK, Fushimi K, Sanui M (2015). Carperitide increases the need for renal replacement therapy after cardiovascular surgery. J Cardiothorac Vasc Anesth.

[62] Mizuno A, Iguchi H, Sawada Y, Hurley M, Nomura H, Hayashi K, Tokuda Y, Watanabe S, Yoshikawa A (2017). The impact of carperitide usage on the cost of hospitalization and outcome in patients with acute heart failure: high value care vs low value care campaign in Japan. Int J Cardiol.

