# Supplementary material for: Low-dose atrial natriuretic peptide for prevention or treatment of acute kidney injury: a systematic review and meta-analysis
Source: Crit Care. 2019 Feb 11;23:41. doi: 10.1186/s13054-019-2330-z (PMC6371622; doi:10.1186/s13054-019-2330-z)
Supplement: Supplementary file 1 — Table S1. PICO model. Table S2. PRISMA-P 2015 Checklist. Table S3. AMSTAR Checklist. Table S4. Terms used to search the electronic databases. Table S5. Excluded studies with full-text reading. Table S6. Sensitivity analysis of incidence of acute kidney injury in the prevention trials. Table S7. Sensitivity analysis of peak serum creatinine in the prevention trials. Table S8. Subgroup analysis. Table S9. GRADE pro summary of finding tables for the prevention of AKI. Table S10. GRADE pro summary of finding tables for the treatment of AKI. Figure S1. Meta-regression results for the reduction of acute kidney injury by low-dose ANP. Figure S2. Trial sequential analysis: acute kidney injury in the prevention trials (random-effects D-L). Figure S3. Trial sequential analysis of acute kidney injury in the prevention trials (random-effects S-J). Figure S4. Trial sequential analysis of acute kidney injury in the prevention trials (random-effects D-L). Figure S5. Trial sequential analysis of renal replacement therapy in the prevention trials (random-effects D-L). Figure S6. Forest plot for hospital stay and ICU stay in the prevention trials. Figure S7. Trial sequential analysis: hospital stay in the prevention trials (random-effects D-L). Figure S8. Forest plot for occurrence of hypotension in the prevention trials. Figure S9. Forest plot for peak serum creatinine in the prevention trials. Figure S10. Forest plot for ICU stay in the treatment trials. Figure S11. Forest plot for occurrence of hypotension in the treatment trials. Figure S12. Trial sequential analysis of renal replacement therapy in the treatment trials. Figure S13. Trial sequential analysis of ICU stay in the treatment trials. Figure S14. Trial sequential analysis of occurrence of hypotension in the treatment trials. Figure S15. Funnel plots in the prevention trials. Figure S16. Funnel plots in the treatment trials. (DOCX 60973 kb) [file 13054_2019_2330_MOESM1_ESM.docx]

**Supplementary materials**

**Low-dose atrial natriuretic peptide for prevention or treatment of acute kidney injury: a systematic review and meta-analysis**

**Summary**

**page No.**

Supplementary Table 1: PICO model 3

Supplementary Table 2: PRISMA-P 2015 Checklist 4

Supplementary Table 3: AMSTAR Checklist 6

Supplementary Table 4: Terms used to search the electronic databases 9

Supplementary Table 5: Excluded studies with full-text reading 12

Supplementary Table 6: Sensitivity analysis of incidence of acute kidney injury in the prevention trials 13

Supplementary Table 7: Sensitivity analysis of peak serum creatinine in the prevention trials 14

Supplementary Table 8: Subgroup analysis 15

Supplementary Table 9: GRADE pro summary of finding tables for the prevention of AKI 18

Supplementary Table 10: GRADE pro summary of finding tables for the treatment of AKI 19

Supplementary Figure 1: Meta-regression results for the reduction of acute kidney injury by low-dose ANP 20

Supplementary Figure 2: Trial sequential analysis: acute kidney injury in the prevention trials (random-effects D-L) 21

Supplementary Figure 3: Trial sequential analysis of acute kidney injury in the prevention trials (random-effects S-J) 22

Supplementary Figure 4: Trial sequential analysis of acute kidney injury in the prevention trials (random-effects D-L) 23

Supplementary Figure 5: Trial sequential analysis of renal replacement therapy in the prevention trials (random-effects D-L) 24

Supplementary Figure 6: Forest plot for hospital stay and ICU stay in the prevention trials 25

Supplementary Figure 7: Trial sequential analysis: hospital stay in the prevention trials (random-effects D-L) 26

Supplementary Figure 8: Forest plot for occurrence of hypotension in the prevention trials 27

Supplementary Figure 9: Forest plot for peak serum creatinine in the prevention trials28

Supplementary Figure 10: Forest plot for ICU stay in the treatment trials 29

Supplementary Figure 11: Forest plot for occurrence of hypotension in the treatment trials 30

Supplementary Figure 12: Trial sequential analysis of renal replacement therapy in the

treatment trials 31

Supplementary Figure 13: Trial sequential analysis of ICU stay in the treatment trials 32

Supplementary Figure 14: Trial sequential analysis of occurrence of hypotension in the

treatment trials 33

Supplementary Figure 15: Funnel plots in the prevention trials 34

Supplementary Figure 16: Funnel plots in the treatment trials 35

**Table S1: PICO model**

| **P** (Patients) | Patients with or at risk of AKI |
| --- | --- |
| **I** (Intervention) | Administration of low dose ANP (≦ 50ng/kg/min) |
| **C** (Comparator) | Patients who receive placebo or conventional therapy |
| **O** (Outcome) | Incidence of AKI, in-hospital mortality, Requirement for renal replacement therapy, Length of ICU stay, Length of Hospital stay, Occurrence of hypotension, Peak serum creatinine |

**Table S2: PRISMA-P 2015 Checklist**

| **Section/topic** | **#** | **Checklist item** | **Reported on page no.** |
| --- | --- | --- | --- |
|  |  |  |  |
| **ADMINISTRATIVE INFORMATION** | | | |
| **Title** | | | |
| Identification | 1a | Identify the report as a protocol of a systematic review | 8 |
| Update | 1b | If the protocol is for an update of a previous systematic review, identify as such | 8 |
| **Registration** | 2 | If registered, provide the name of the registry (e.g., PROSPERO) and registration number in the Abstract | 5 |
| **Authors** | | | |
| Contact | 3a | Provide name, institutional affiliation, and e-mail address of all protocol authors; provide physical mailing address of corresponding author | 1-3 |
| Contributions | 3b | Describe contributions of protocol authors and identify the guarantor of the review | N/A |
| **Amendments** | 4 | If the protocol represents an amendment of a previously completed or published protocol, identify as such and list changes; otherwise, state plan for documenting important protocol amendments | mentioned in PROSPERO protocol no. CRD42017068568 |
| **Support** | | | |
| Sources | 5a | Indicate sources of financial or other support for the review | 22, 23 |
| Sponsor | 5b | Provide name for the review funder and/or sponsor | N/A |
| Role of sponsor/funder | 5c | Describe roles of funder(s), sponsor(s), and/or institution(s), if any, in developing the protocol | N/A |
| **INTRODUCTION** | | | |
| **Rationale** | 6 | Describe the rationale for the review in the context of what is already known | 6,7 |
| **Objectives** | 7 | Provide an explicit statement of the question(s) the review will address with reference to participants, interventions, comparators, and outcomes (PICO) | Supplementary Table 1 |
| **METHODS** | | | |
| **Eligibility criteria** | 8 | Specify the study characteristics (e.g., PICO, study design, setting, time frame) and report characteristics (e.g., years considered, language, publication status) to be used as criteria for eligibility for the review | 8-10 and Supplementary Table 1 |
| **Information sources** | 9 | Describe all intended information sources (e.g., electronic databases, contact with study authors, trial registers, or other grey literature sources) with planned dates of coverage | 8,9 |
| **Search strategy** | 10 | Present draft of search strategy to be used for at least one electronic database, including planned limits, such that it could be repeated | 8,9 |
| ***STUDY RECORDS*** | | | |
| Data management | 11a | Describe the mechanism(s) that will be used to manage records and data throughout the review | 8-11 |
| Selection process | 11b | State the process that will be used for selecting studies (e.g., two independent reviewers) through each phase of the review (i.e., screening, eligibility, and inclusion in meta-analysis) | 9 |
| Data collection process | 11c | Describe planned method of extracting data from reports (e.g., piloting forms, done independently, in duplicate), any processes for obtaining and confirming data from investigators | 10 |
| **Data items** | 12 | List and define all variables for which data will be sought (e.g., PICO items, funding sources), any pre-planned data assumptions and simplifications | 9-10 and Supplementary Table 1 |
| **Outcomes and prioritization** | 13 | List and define all outcomes for which data will be sought, including prioritization of main and additional outcomes, with rationale | 9-10 and Supplementary Table 10 and 11 |
| **Risk of bias in individual studies** | 14 | Describe anticipated methods for assessing risk of bias of individual studies, including whether this will be done at the outcome or study level, or both; state how this information will be used in data synthesis | 10-12 |
| ***DATA*** | | | |
| **Synthesis** | 15a | Describe criteria under which study data will be quantitatively synthesized | 9-11 |
|  | 15b | If data are appropriate for quantitative synthesis, describe planned summary measures, methods of handling data, and methods of combining data from studies, including any planned exploration of consistency (e.g., *I* ^2^, Kendall’s tau) | 9-12 |
|  | 15c | Describe any proposed additional analyses (e.g., sensitivity or subgroup analyses, meta-regression) | 11-12 |
|  | 15d | If quantitative synthesis is not appropriate, describe the type of summary planned | N/A |
| **Meta-bias(es)** | 16 | Specify any planned assessment of meta-bias(es) (e.g., publication bias across studies, selective reporting within studies) | 10-11 |
| **Confidence in cumulative evidence** | 17 | Describe how the strength of the body of evidence will be assessed (e.g., GRADE) | 12-13 |

**Table S3: AMSTAR checklist**

**
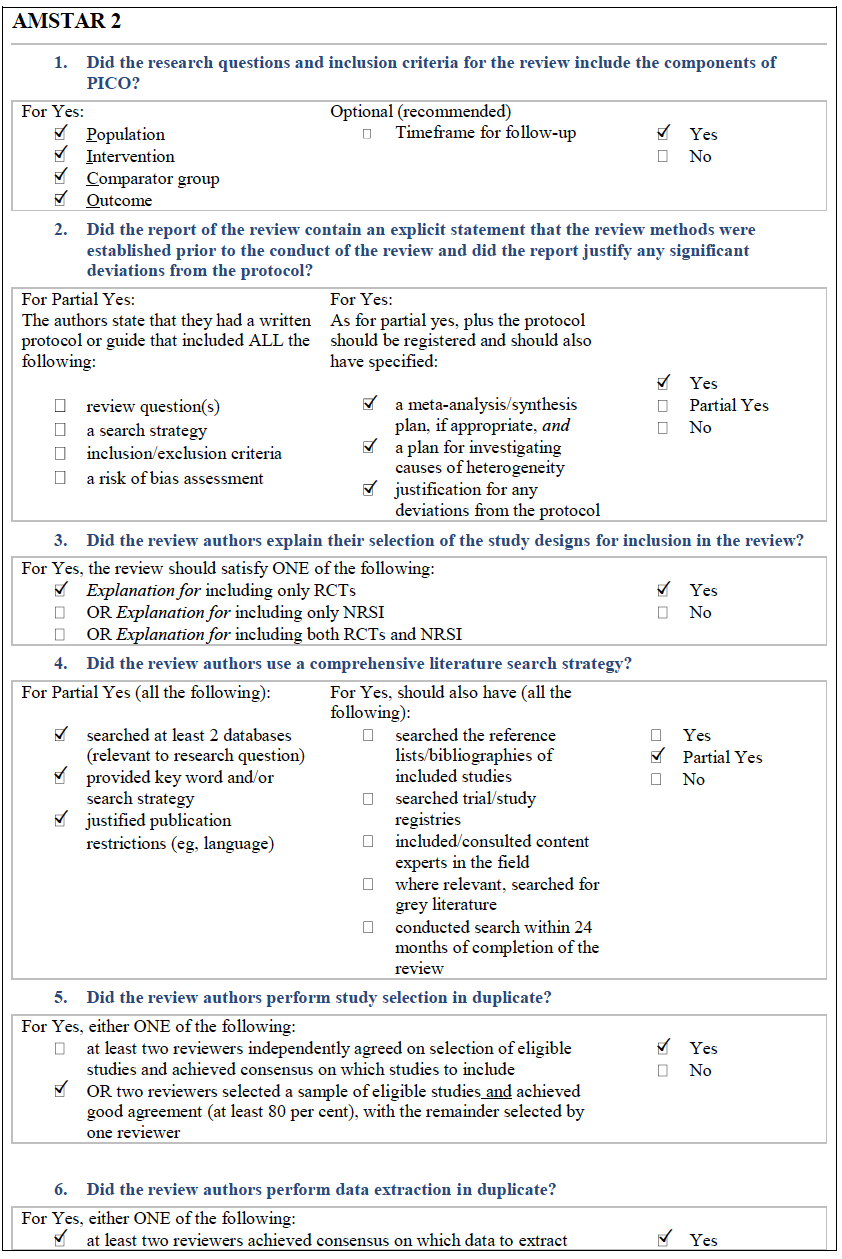
**

**
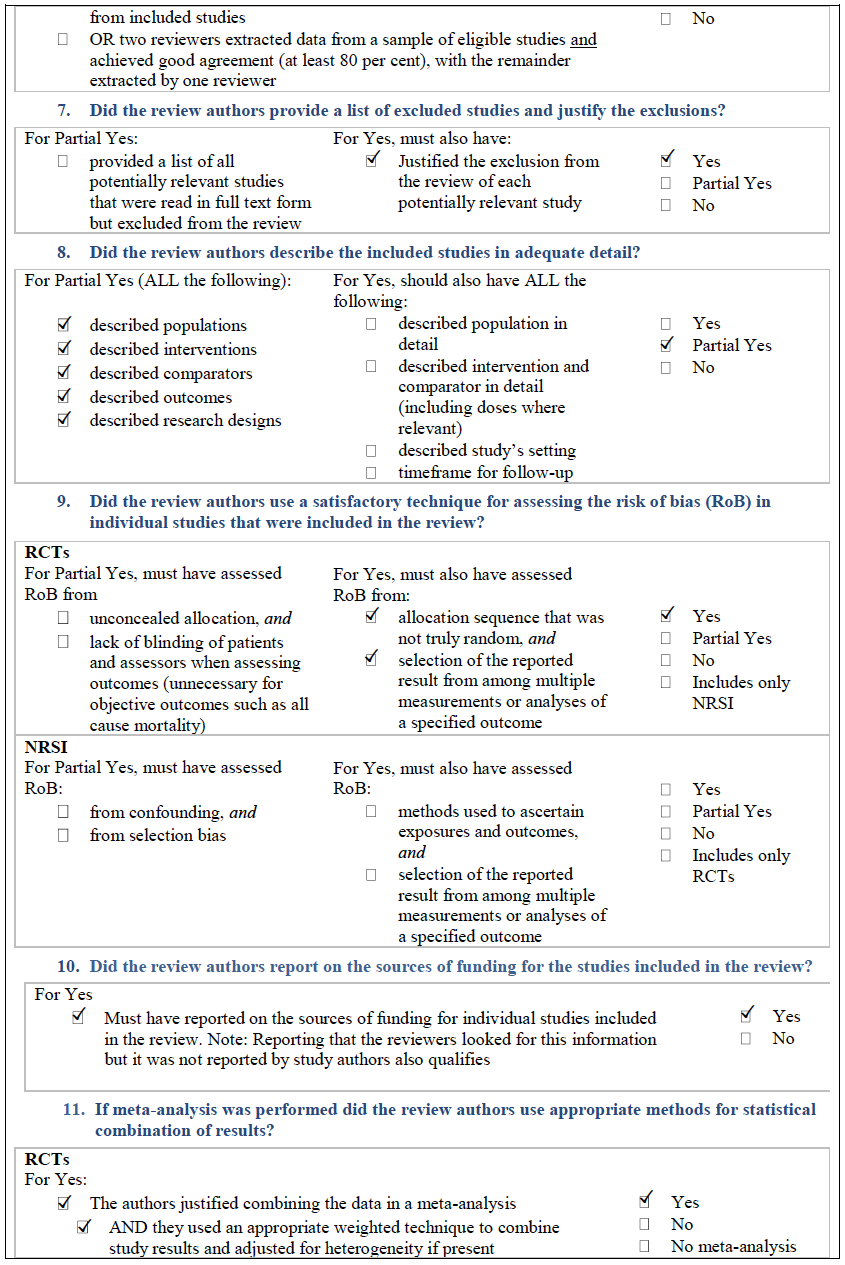
**

**
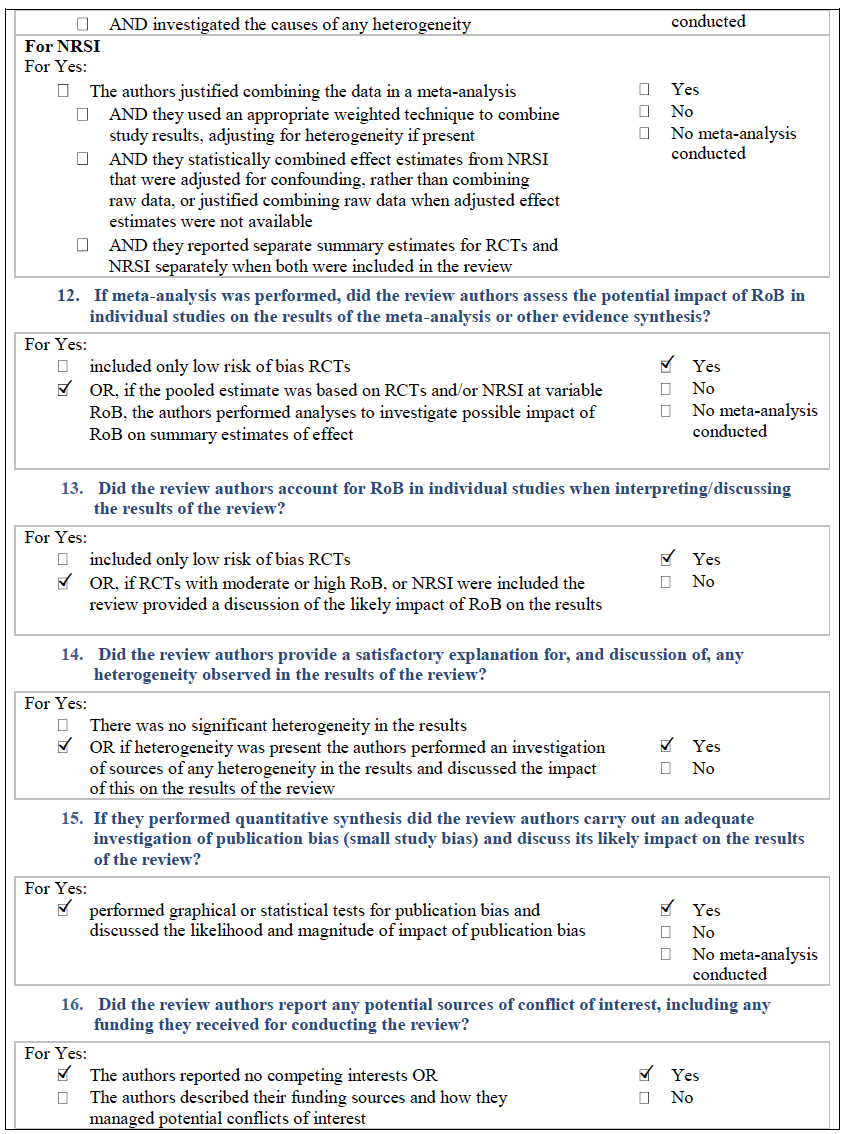
**

**Table S4: Terms used to search the electronic databases**

| **Electric database** | **Search terms** | **Hits** |
| --- | --- | --- |
| Medline (Pubmed) | #1. exp Acute Kidney Injury/  #2. (acute kidney failure or acute renal failure).tw.  #3. (acute kidney injur$ or acute renal injur$).tw  #4. (acute kidney insufficie$ or acute renal insufficie$).tw.  #5. acute tubular necrosis.tw.  #6. (ARI or AKI or ARF or AKF or ATN).tw.  #7. or/1-6  #8 exp Atrial Natriuretic Factor/  #9 (atrial natriuretic peptide$ or ANP or ANF).tw.  #10. carperitide.tw.  #11. atriopeptin$.tw.  #12. anaritide$.tw.  #13. (natriuretic$ and (peptide$ or factor$)).tw.  #14. hANP.tw.  #15. or/8-14  #16. 7 and 15 | 41339  20953  12338  1705  2864  19313  61821  14857  15632  98  568  20  29544  643  33686  410 |
| Embase | #1. ‘atrial natriuretic factor’  #2. atrial AND natriuretic AND peptide  #3. anp  #4. anf  #5. anaritide  #6. atriopeptin  #7. carperitide  #8. nppa AND protein  #9. #1 or #2 or #3 or #4 or #5 or #6 or #7 or #8  #10. acute AND kidney AND injury  #11. acute AND kidney AND failure  #12. acute AND renal AND failure  #13. aki  #14. worsening AND renal AND function  #15. acute AND renal AND insufficiency  #16. acute AND kidney AND insufficiency  #17. arf  #18. acute AND tubular AND necrosis  #19. acute AND kidney AND tubular AND necrosis  #20. atn  #21. ‘acute kidney failure’  #22. ‘kidney failure’  #23. #10 or #11 or #12 or #13 or #14 or #15 or #16 or #17 or #18 or #19 or #20 or #21 or #22  #24. #9 AND #23 | 21724  19591  13944  4661  147  723  231  219  35008  42420  111966  83893  21552  4339  8747  8683  13437  6561  6313  2400  66911  290554  349303  2101 |
| Cochrane Library | #1. MeSH descriptor: [Atrial Natriuretic Factor] explode all trees  #2. atrial natriuretic factor  #3. atrial natriuretic peptide  #4. anp  #5. anf  #6. anaritide  #7. atriopeptin  #8. carperitide  #9. nppa AND protein  #10. #1 or #2 or #3 or #4 or #5 or #6 or #7 or #8 or #9  #11. MeSH descriptor: [Acute Kidney Injury] explode all trees  #12. acute kidney injury  #13. acute kidney failure  #14. acute renal failure  #15. AKI  #16. worsening renal failure  #17. acute renal insufficiency  #18. acute kidney insufficiency  #19. ARF  #20. acute tubular necrosis  #21. acute kidney tubular necrosis  #22. atn  #23. MeSH descriptor: [Renal Insufficiency] explode all trees  #24. #11 or #12 or #13 or #14 or #15 or #16 or #17 or #18 or #19 or #20 or #21  or #22 or #23  #25. #10 and #24 | 862  1329  1379  618  213  19  5  32  2  1882  965  2568  4555  5029  774  839  1085  688  399  191  173  150  6164  12497  181 |

**Table S5: Excluded studies with full-text reading**

| Excluded studies | Reasons |
| --- | --- |
| Woolf AS, et al. 1989 [1] | Not a randomized controlled trial |
| Kurnik BR, et al. 1990 [2] | Control group was treated with mannitol |
| Ratcliffe PJ, et al. 1991 [3] | This study is for cadaveric kidney |
| Sands JM, et al. 1991 [4] | This study is for cadaveric kidney |
| Lang CC, et al. 1992 [5] | There is no relevant data for this review |
| Rahman SN, et al. 1994 [6] | The dose of ANP is too high |
| Bergman A, et al. 1996 [7] | The dose of ANP is too high |
| Allgren RL, et al. 1997 [8] | The dose of ANP is too high |
| Lewis J, et al. 2000 [9] | The dose of ANP is too high |
| Sezai A, et al. 2000 [10] | It has potential patient overlap |
| Akamatsu N, et al. 2005 [11] | The dose of ANP is too high |
| Sezai A, et al. 2006 [12] | Not a randomized controlled trial |
| Sezai A, et al. 2006 [13] | It has potential patient overlap |
| Sezai A, et al. 2007 [14] | It has potential patient overlap |
| Sezai A, et al. 2010 [15] | It has potential patient overlap |
| Yoshitake I et al. 2011 [16] | Not a randomized controlled trial |
| Sezai A, et al. 2013 [17] | Not a randomized controlled trial (subgroup analysis) |
| Sezai A, et al. 2014 [18] | Not a randomized controlled trial (subgroup analysis) |
| Suzuki S, et al. 2014 [19] | Control group was treated with tolvaptan |
| Wang G, et al. 2016 [20] | The dose of ANP is too high |
| Takaya Y, et al. 2017 [21] | Not a randomized controlled trial |
| Tsukamoto M, et al. 2017 [22] | There is no relevant data for this review |
|  |  |
| Abbreviations: AKI, acute kidney injury; ANP, atrial natriuretic peptide | |

**Table S6: Sensitivity analysis of induction of AKI in the prevention trials**

| Omitted Study | Participants | Effect estimate | p value  for heterogeneity | Heterogeneity |
| --- | --- | --- | --- | --- |
| Kurnik BR et al. 1998 [23] | 187 | 0.44 ( 0.35 to 0.55 ) | 0.37 | I^2^=7% |
| Morikawa S et al. 2009 [24] | 252 | 0.54 ( 0.38 to 0.78 ) | 0.05 | I^2^=53% |
| Sezai A et al. 2009 [25] | 504 | 0.55 ( 0.34 to 0.88 ) | 0.04 | I^2^=55% |
| Tamura Y et al. 2009 [26] | 39 | 0.50 ( 0.35 to 0.70 ) | 0.06 | I^2^=51% |
| Sezai A et al. 2011 [27] | 285 | 0.55 ( 0.34 to 0.89 ) | 0.04 | I^2^=55% |
| Okumura N et al. 2012 [28] | 112 | 0.48 ( 0.35 to 0.67 ) | 0.08 | I^2^=46% |
| Mori Y et al. 2014 [29] | 42 | 0.53 ( 0.36 to 0.80 ) | 0.03 | I^2^=56% |
| Moriyama T et al. 2017 [30] | 48 | 0.54 ( 0.38 to 0.76 ) | 0.05 | I^2^=53% |

**Table S7: Sensitivity analysis of peak serum creatinine in the prevention trials**

| Omitted Study | Participants | Effect estimate | p value  for heterogeneity | Heterogeneity |
| --- | --- | --- | --- | --- |
| Mitaka et al. 2008 [31] | 40 | -0.14 ( -0.35 to 0.07 ) | 0.006 | I^2^=81% |
| Sezai A et al. 2009 [25] | 506 | -0.24 ( -0.64 to 0.17 ) | 0.004 | I^2^=82% |
| Sezai A et al. 2011 [27] | 285 | -0.13 ( -0.36 to 0.11 ) | 0.008 | I^2^=79% |
| Tamura Y et al. 2011 [26] | 39 | -0.28 ( -0.48 to -0.07 ) | 0.11 | I^2^=54% |

**Table S8: Subgroup analysis**

| Outcome or subgroup | Studies | Participants | Statistical method | Effect estimate | p value | Heterogeneity |
| --- | --- | --- | --- | --- | --- | --- |
| **Cardiovascular surgery** |  |  |  |  |  |  |
| acute kidney injury | 4 | 869 | Risk ratio (M-H, random, 95% CI) | 0.43 (0.34 to 0.53) | <0.0001 | I^2^=0% |
| in-hospital mortality | 7 | 987 | Risk ratio (M-H, random, 95% CI) | 0.49 (0.17 to 1.44) | 0.14 | I^2^=0% |
| renal replacement therapy | 7 | 1009 | Risk ratio (M-H, random, 95% CI) | 0.14 (0.03 to 0.63) | 0.005 | I^2^=0% |
| hospital stay | 4 | 849 | Mean difference (IV, random, 95% CI) | -2.90 (-3.00 to -2.80) | <0.0001 | I^2^=44% |
| ICU stay | 3 | 78 | Mean difference (IV, random, 95% CI) | 0.03 (-0.46 to 0.52) | 0.25 | I^2^=64% |
| occurrence of hypotension | 9 | 1080 | Peto Odds ratio (Peto, fixed, 95% CI) | Not estimable | Not applicable | Not applicable |
| peak serum creatinine | 3 | 829 | Mean difference (IV, random, 95% CI) | -0.28 (-0.48 to -0.07) | 0.008 | I^2^=54% |
| **Contrast medium** |  |  |  |  |  |  |
| acute kidney injury | 3 | 553 | Risk ratio (M-H, random, 95% CI) | 0.75 (0.29 to 1.97) | 0.26 | I^2^=65% |
| in-hospital mortality | 0 | 0 | Risk ratio (M-H, random, 95% CI) | Not estimable | Not applicable | Not applicable |
| renal replacement therapy | 1 | 254 | Risk ratio (M-H, random, 95% CI) | 0.34 (0.01 to 8.23) | 0.51 | Not applicable |
| hospital stay | 0 | 0 | Mean difference (IV, random, 95% CI) | Not estimable | Not applicable | Not applicable |
| ICU stay | 0 | 0 | Mean difference (IV, random, 95% CI) | Not estimable | Not applicable | Not applicable |
| occurrence of hypotension | 1 | 112 | Peto Odds ratio (Peto, fixed, 95% CI) | 10.23 (4.55 to 23.00) | <0.0001 | Not applicable |
| Peak serum creatinine | 0 | 0 | Mean difference (IV, random, 95% CI) | Not estimable | Not applicable | Not applicable |
| **Placebo** |  |  |  |  |  |  |
| acute kidney injury | 7 | 1432 | Risk ratio (M-H, random, 95% CI) | 0.50 (0.35 to 0.70) | <0.0001 | I^2^=51% |
| in-hospital mortality | 3 | 831 | Risk ratio (M-H, random, 95% CI) | 0.49 (0.17 to 1.44) | 0.20 | I^2^=0% |
| renal replacement therapy | 3 | 336 | Risk ratio (M-H, random, 95% CI) | 0.34 (0.01 to 8.23) | 0.51 | Not applicable |
| hospital stay | 3 | 831 | Mean difference (IV, random, 95% CI) | -2.90 (-3.00 to -2.80) | <0.0001 | I^2^=0% |
| ICU stay | 1 | 42 | Mean difference (IV, random, 95% CI) | 0.30 (-0.67 to 1.27) | 0.54 | Not applicable |
| occurrence of hypotension | 6 | 1036 | Peto Odds ratio (Peto, fixed, 95% CI) | 10.23 (4.55 to 23.00) | <0.0001 | Not applicable |
| Peak serum creatinine | 7 | 829 | Mean difference (IV, random, 95% CI) | -0.28 (-0.48 to -0.07) | 0.008 | I^2^=54% |
| **Control** |  |  |  |  |  |  |
| acute kidney injury | 1 | 39 | Risk ratio (M-H, random, 95% CI) | 2.11 (0.21 to 21.36) | 0.53 | Not applicable |
| in-hospital mortality | 5 | 180 | Risk ratio (M-H, random, 95% CI) | 0.11 (0.01 to 2.02) | 0.14 | Not applicable |
| renal replacement therapy | 5 | 927 | Risk ratio (M-H, random, 95% CI) | 0.14 (0.03 to 0.63) | 0.01 | I^2^=0% |
| hospital stay | 3 | 106 | Mean difference (IV, random, 95% CI) | 5.00 (-5.94 to 15.95) | 0.37 | I^2^=55% |
| ICU stay | 3 | 75 | Mean difference (IV, random, 95% CI) | 0.07 (-0.41 to 0.55) | 0.76 | I^2^=41% |
| occurrence of hypotension | 5 | 180 | Peto Odds ratio (Peto, fixed, 95% CI) | 0.14 (0.00 to 6.82) | 0.32 | Not applicable |
| peak serum creatinine | 4 | 868 | Mean difference (IV, random, 95% CI) | -0.18 (-0.38 to 0.01) | Not applicable | Not applicable |
| **ANP administration time > 24 hours** |  |  |  |  |  |  |
| acute kidney injury | 6 | 1172 | Risk ratio (M-H, random, 95% CI) | 0.42 (0.34 to 0.52) | <0.0001 | I^2^=0% |
| in-hospital mortality | 6 | 969 | Risk ratio (M-H, random, 95% CI) | 0.49 (0.17 to 1.44) | 0.20 | I^2^=0% |
| renal replacement therapy | 8 | 1263 | Risk ratio (M-H, random, 95% CI) | 0.17 (0.04 to 0.64) | 0.009 | I^2^=0% |
| hospital stay | 5 | 898 | Mean difference (IV, random, 95% CI) | -2.80 (-4.53 to -1.08) | 0.001 | I^2^=39% |
| ICU stay | 2 | 69 | Mean difference (IV, random, 95% CI) | 0.46 (-0.28 to 1.26) | 0.22 | I^2^=0% |
| occurrence of hypotension | 7 | 1017 | Peto Odds ratio (Peto, fixed, 95% CI) | Not estimable | Not applicable | Not applicable |
| peak serum creatinine | 3 | 829 | Mean difference (IV, random, 95% CI) | -0.28 (-0.48 to -0.07) | 0.008 | I^2^=54% |
| **ANP administration time < 24 hours** |  |  |  |  |  |  |
| acute kidney injury | 2 | 151 | Risk ratio (M-H, random, 95% CI) | 1.64 (0.50 to 5.37) | 0.42 | I^2^=0% |
| in-hospital mortality | 2 | 42 | Risk ratio (M-H, random, 95% CI) | Not estimable | Not applicable | Not applicable |
| renal replacement therapy | 0 | 0 | Risk ratio (M-H, random, 95% CI) | Not estimable | Not applicable | Not applicable |
| hospital stay | 1 | 39 | Mean difference (IV, random, 95% CI) | 6.40 (-8.84 to 21.64) | 0.41 | Not applicable |
| ICU stay | 2 | 57 | Mean difference (IV, random, 95% CI) | -0.04 (-0.50 to 0.41) | 0.85 | I^2^=41% |
| occurrence of hypotension | 4 | 199 | Peto Odds ratio (Peto, fixed, 95% CI) | 2.82 (0.07 to 106.08) | 0.58 | I^2^=78% |
| peak serum creatinine | 1 | 18 | Mean difference (IV, random, 95% CI) | 0.09 (-0.09 to 0.27) | 0.33 | Not applicable |
| **Removal of 2 RCTs from an influential group**  **(Sezai et al 2009 and Sezai et al 2011)** |  |  |  |  |  |  |
| acute kidney injury | 6 | 682 | Risk ratio (M-H, random, 95% CI) | 0.60 (0.31 to 1.15) | 0.05 | I^2^=55% |
| in-hospital mortality | 6 | 222 | Risk ratio (M-H, random, 95% CI) | 0.19 (0.02 to 1.62) | 0.58 | I^2^=0% |
| renal replacement therapy | 6 | 474 | Risk ratio (M-H, random, 95% CI) | 0.29 (0.03 to 2.76) | 0.90 | I^2^=0% |
| hospital stay | 4 | 148 | Mean difference (IV, random, 95% CI) | 0.54 (-4.63 to 5.70) | 0.17 | I^2^=41% |
| ICU stay | 4 | 117 | Mean difference (IV, random, 95% CI) | 0.06 (-0.31 to 0.43) | 0.27 | I^2^=23% |
| occurrence of hypotension | 9 | 427 | Peto Odds ratio (Peto, fixed, 95% CI) | 8.57 (3.88 to 18.95) | 0.03 | I^2^=78% |
| peak serum creatinine | 2 | 79 | Mean difference (IV, random, 95% CI) | -0.17 (-0.75 to 0.42) | 0.02 | I^2^=80% |

Abbreviations: ANP, atrial natriuretic peptide; CI, confidence interval; ICU, intensive care unit: IV, inverse variance; M-H, Mantel-Haenszel

**Table S9: GRADE pro summary of finding tables for the prevention of AKI**

**Table S10: GRADE pro summary of finding tables for the treatment of AKI**

**Figure S1: Meta-regression results for the reduction of acute kidney injury by low-dose ANP**

a. Age, b. ANP administration time, c. Cardiopulmonary bypass (CPB), d. Contrast medium

**Figure S2: Trial sequential analysis: acute kidney injury in the prevention trials (α= 5%, β= 80%, RRR=25%, random-effects D-L).**

Complete blue line represents the cumulative Z-curve, complete red line represents the trial sequential monitoring boundary for benefit and dotted green line represents the conventional boundary for benefit. The required information size of 2950 has not been reached. The cumulative Z-curve has crossed both the conventional boundary for benefit and the trial sequential monitoring boundary for benefit.

**
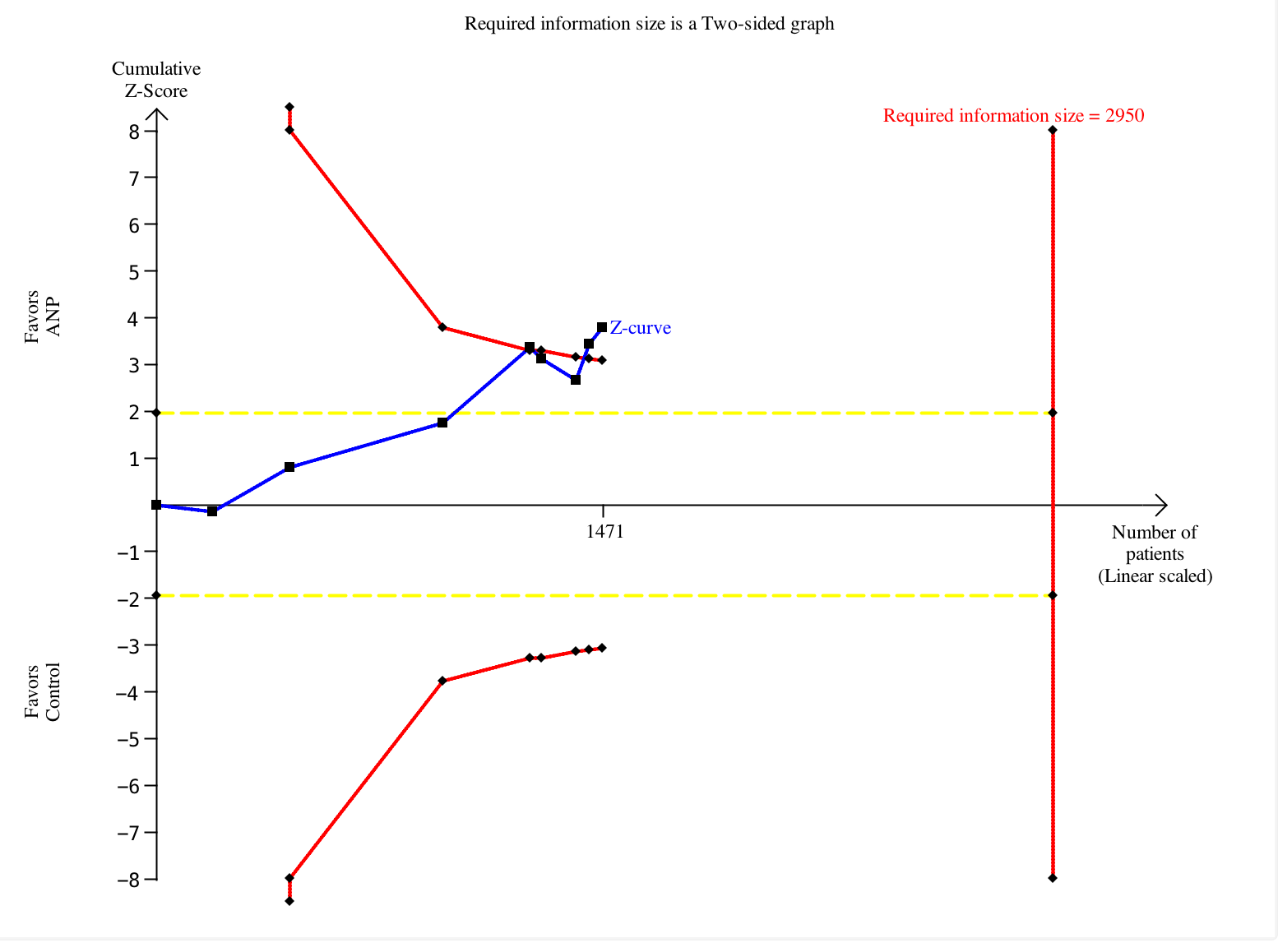
**

**Figure S3: Trial sequential analysis: acute kidney injury in the prevention trials (α= 5%, β= 80%, RRR=25%, random-effects S-J).**

Complete blue line represents the cumulative Z-curve, complete red line represents the trial sequential monitoring boundary for benefit and dotted green line represents the conventional boundary for benefit. The required information size of 6640 has not been reached. The cumulative Z-curve has crossed the conventional boundary for benefit but not the trial sequential monitoring boundary for benefit.

**
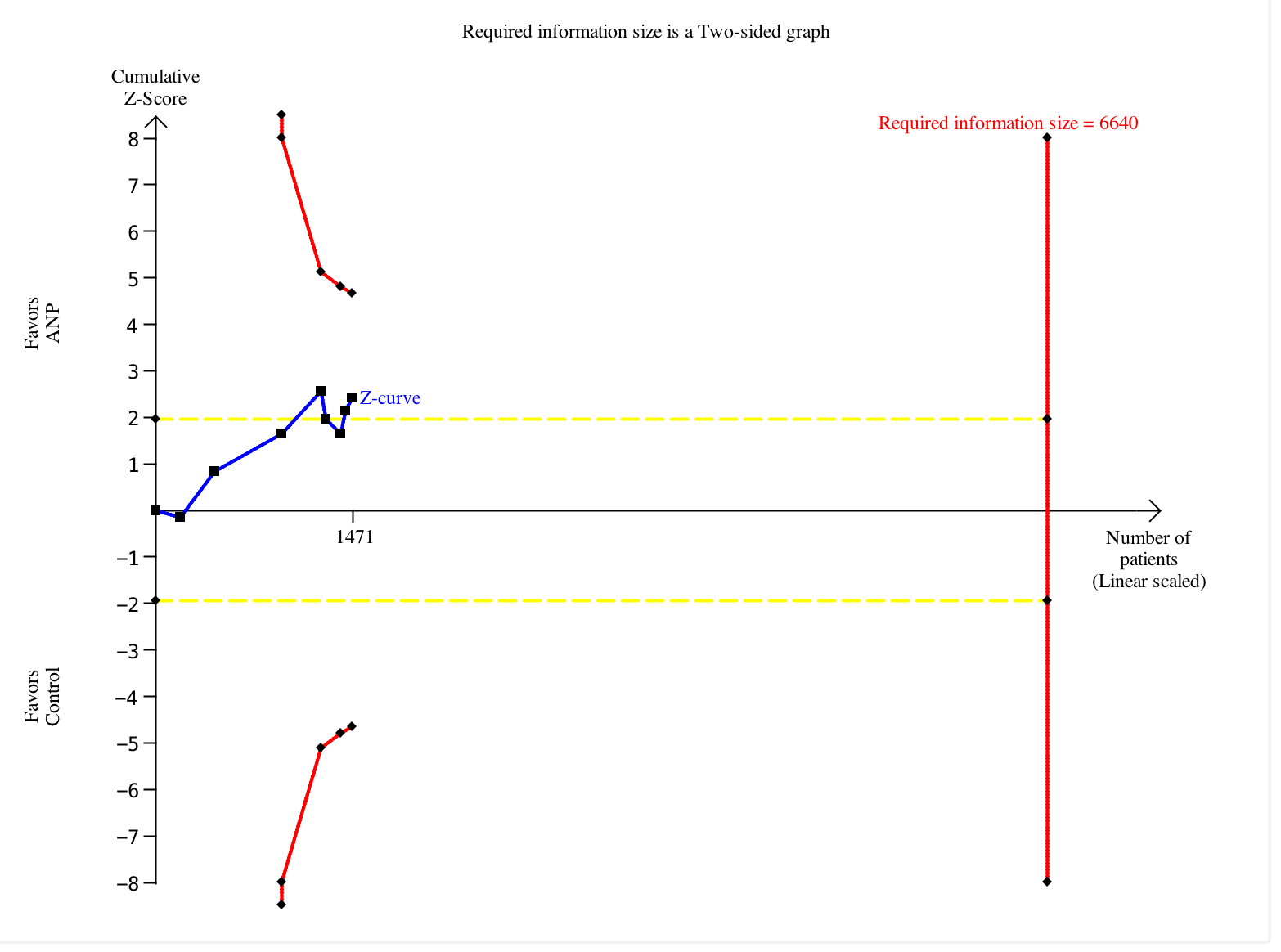
**

**Figure S4: Trial sequential analysis: in-hospital mortality in the prevention trials (α= 5%, β= 80%, RRR = 25%, random-effects D-L).**

Complete blue line represents the cumulative Z-curve, complete red line represents the trial sequential monitoring boundary for benefit and dotted green line represents the conventional boundary for benefit. The required information size of 16712 has not been reached. The cumulative Z-curve has not cross the conventional boundary for benefit or the trial sequential monitoring boundary for benefit.

**
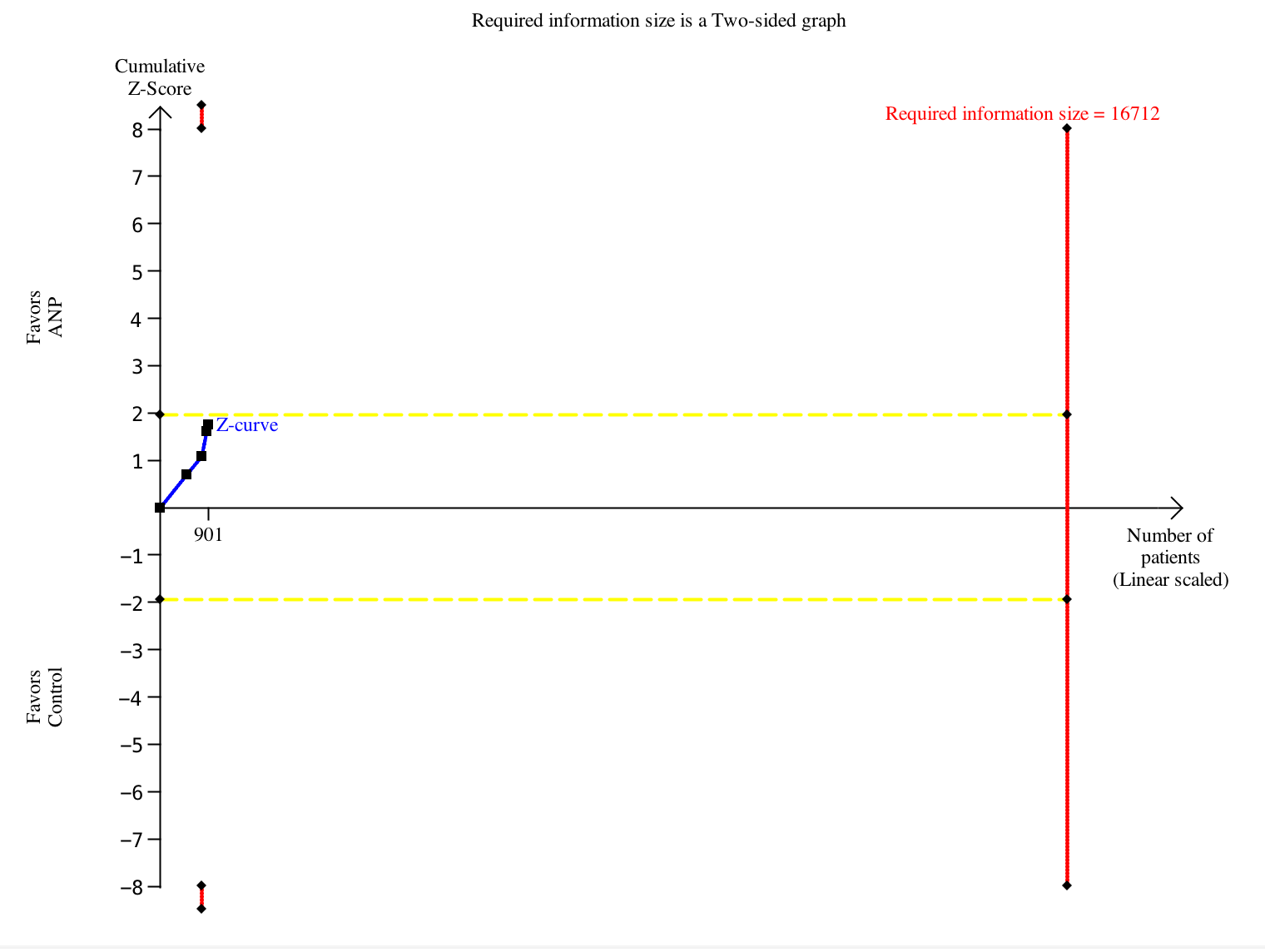
**

**Figure S5: Trial sequential analysis: renal replacement therapy in the prevention trials (α= 5%, β= 80%, RRR = 25%, random-effects D-L).**

Complete blue line represents the cumulative Z-curve, complete red line represents the trial sequential monitoring boundary for benefit and dotted green line represents the conventional boundary for benefit. The required information size of 19595 has not been reached. The cumulative Z-curve has crossed the conventional boundary for benefit but not the trial sequential monitoring boundary for benefit.

**
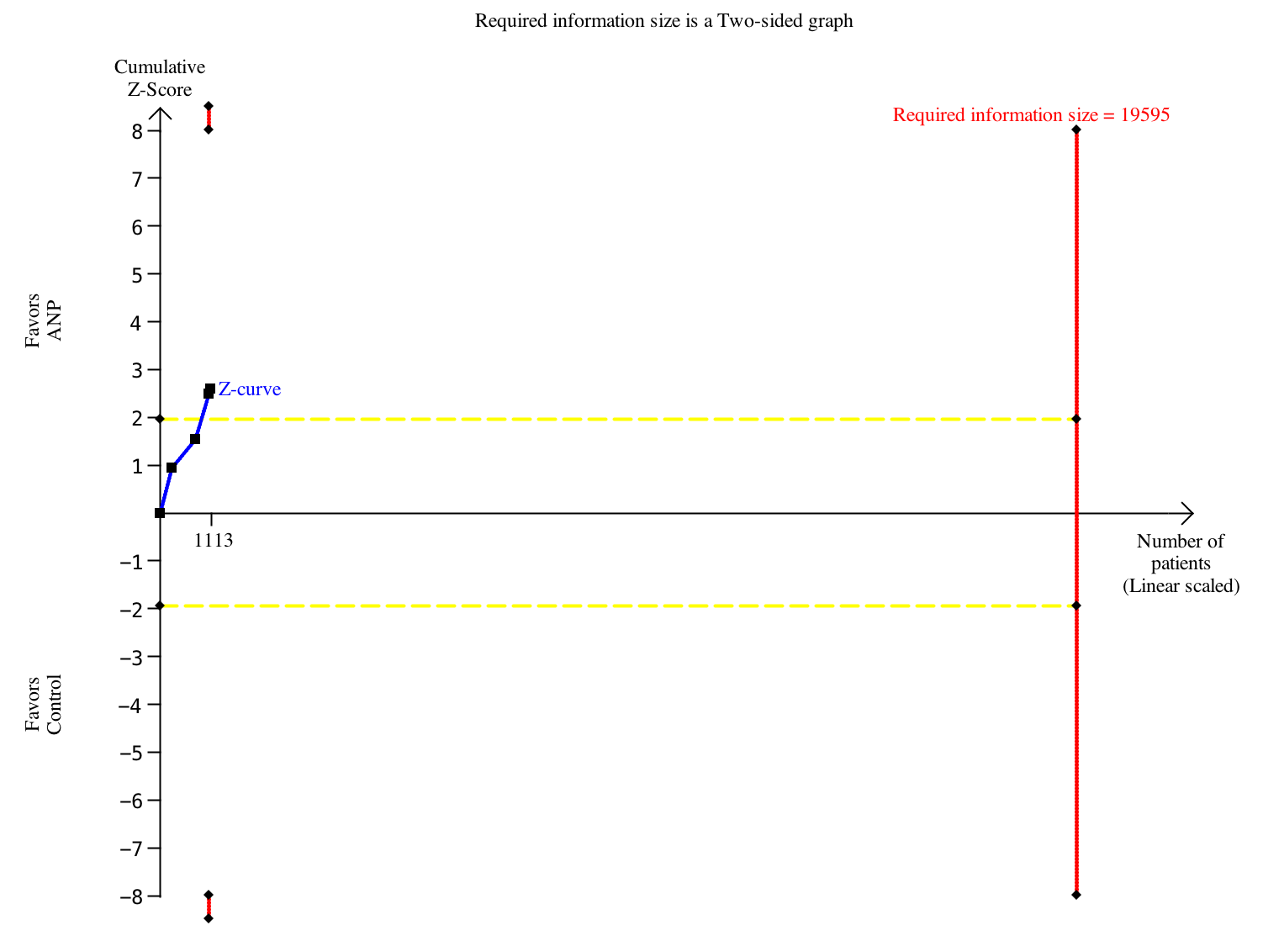
**

**Figure S6: Forest plot for hospital stay and ICU stay in the prevention trials.**

(A) hospital stay and (B) ICU stay. ANP, atrial natriuretic peptide; CI, confidential interval; ICU, intensive care unit; IV, inverse variance; SD, standard deviation.

**A. hospital stay**

**B. ICU stay**

**Figure S7: Trial sequential analysis: hospital stay in the prevention trials (α= 5%, β= 80%, random-effects D-L).**

Complete blue line represents the cumulative Z-curve, complete red line represents the trial sequential monitoring boundary for benefit and dotted green line represents the conventional boundary for benefit. The required information size of 1961 has not been reached. The cumulative Z-curve does cross the conventional boundary for benefit but not the trial sequential monitoring boundary for benefit.


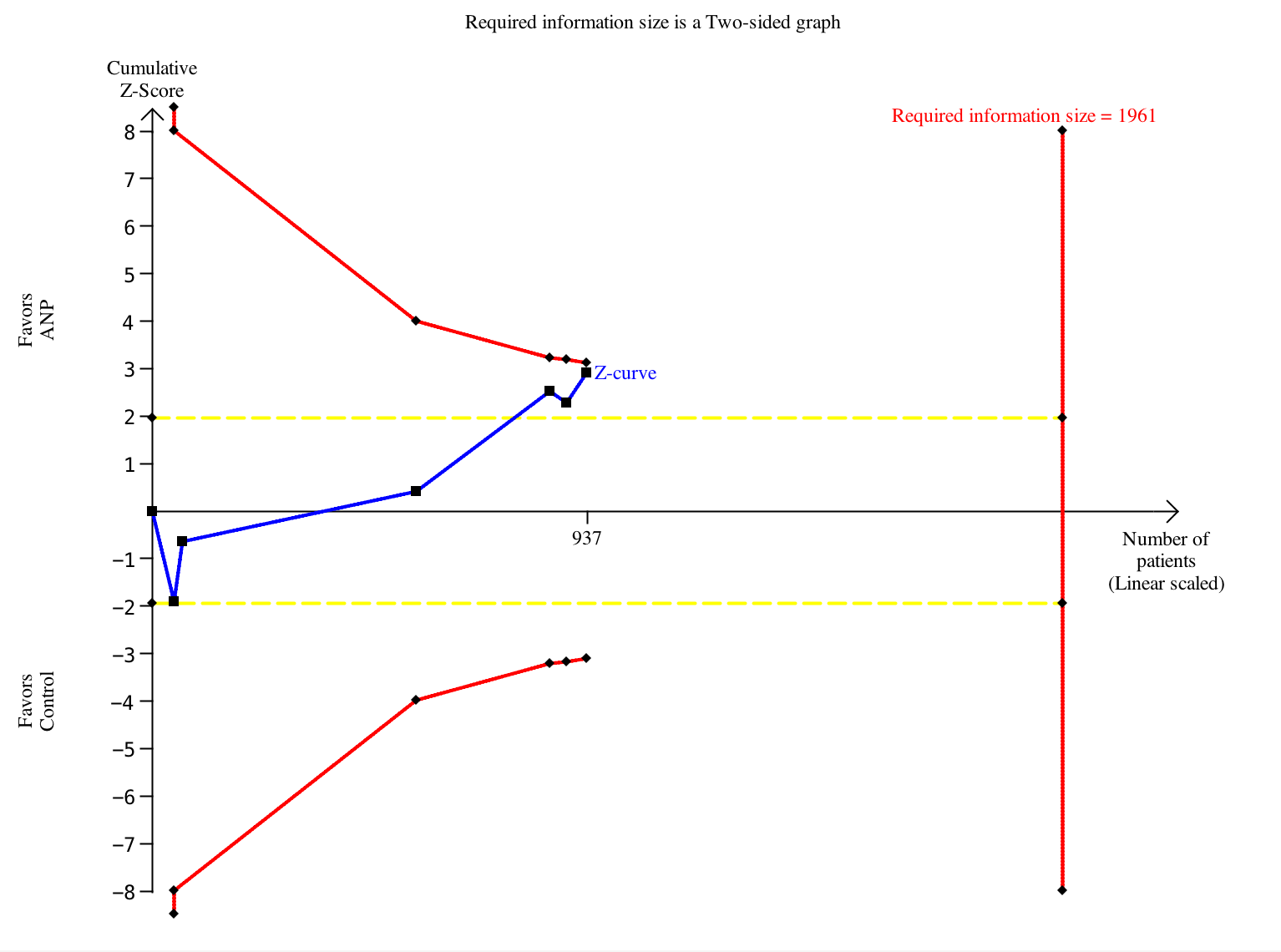


**Figure S8: Forest plot for occurrence of hypotension in the prevention trials.**

ANP, atrial natriuretic peptide; CI, confidential interval;

**Figure S9: Forest plot for peak serum creatinine in the prevention trials.**

ANP, atrial natriuretic peptide; CI, confidential interval; IV, inverse variance; SD, standard deviation.

**Figure S10: Forest plot for ICU stay in the treatment trials**

ANP, atrial natriuretic peptide; CI, confidential interval; ICU, intensive care unit; SD, standard deviation.

**Figure S11: Forest plot for occurrence of hypotension in the treatment trials**

ANP, atrial natriuretic peptide; CI, confidential interval; M-H, Mantel-Haenszel.

**Figure S12: Trial sequential analysis of renal replacement therapy in the treatment trials (α= 5%, β= 80%, RRR = 25%)**

Complete blue line represents the cumulative Z-curve, complete red line represents the trial sequential monitoring boundary for benefit and dotted green line represents the conventional boundary for benefit. The required information size of 1446 has not been reached. The cumulative Z-curve did cross the conventional boundary for benefit but not the trial sequential monitoring boundary for benefit.

**
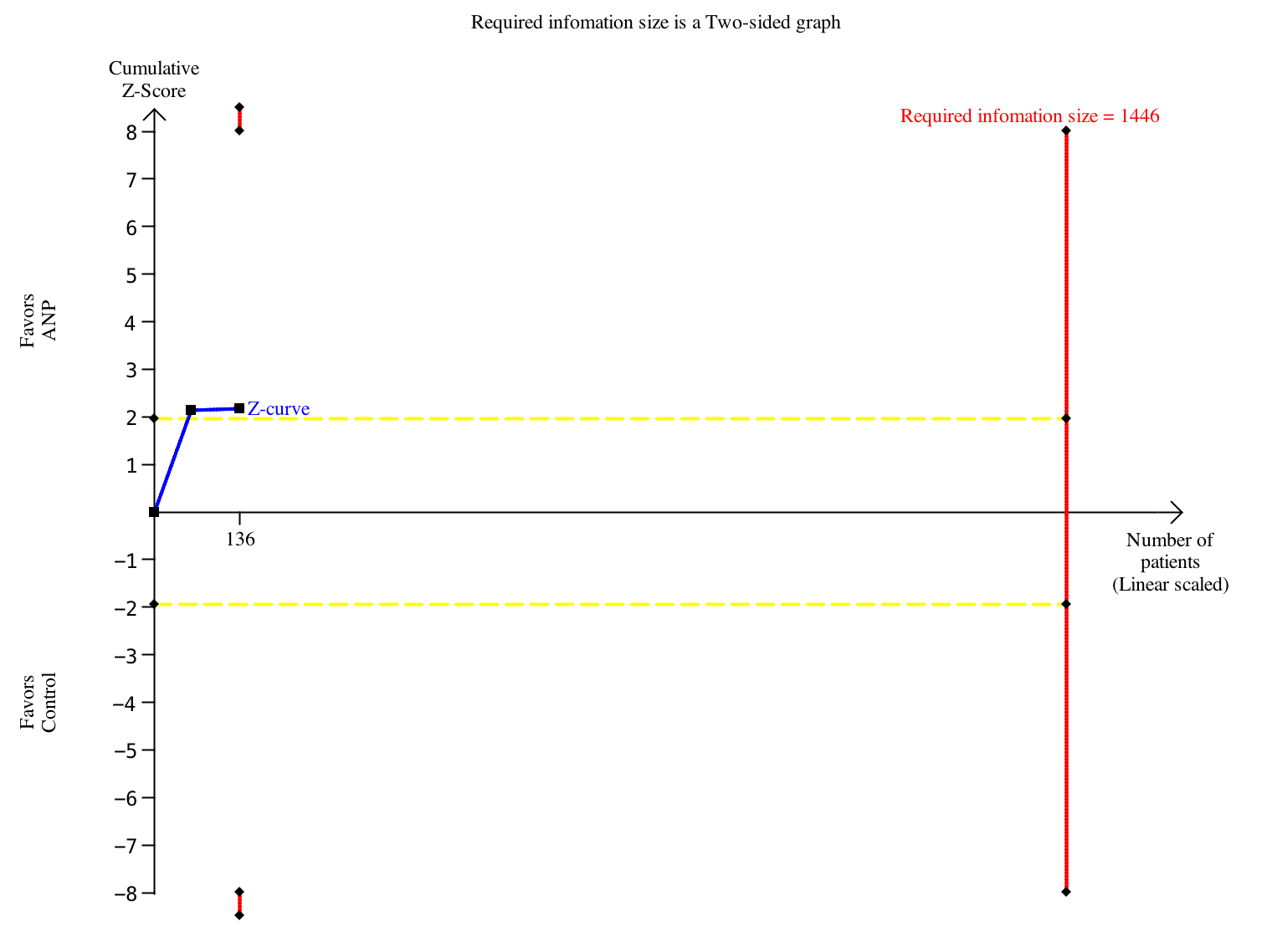
**

**Figure S13: Trial sequential analysis of ICU stay in the treatment trials (α= 5%, β= 80%)**

Complete blue line represents the cumulative Z-curve, complete red line represents the trial sequential monitoring boundary for benefit and dotted green line represents the conventional boundary for benefit. The required information size of 111 has been reached. The cumulative Z-curve crossed both the conventional boundary for benefit and the trial sequential monitoring boundary for benefit. These results indicate that this analysis is conclusive for the positive effect on the outcome.

**
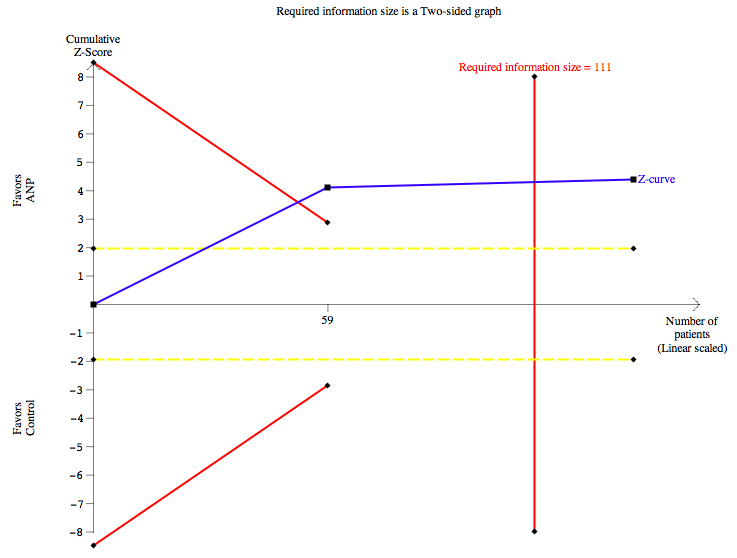
**

**Figure S14: Trial sequential analysis of the occurrence of hypotension in the treatment trials (α= 5%, β= 80%, RRR = 25%)**

Complete blue line represents the cumulative Z-curve, complete red line represents the trial sequential monitoring boundary for benefit and dotted green line represents the conventional boundary for benefit. The required information size of 1670 has not been reached. The cumulative Z-curve does not cross the conventional boundary for benefit or the trial sequential monitoring boundary for benefit.

**
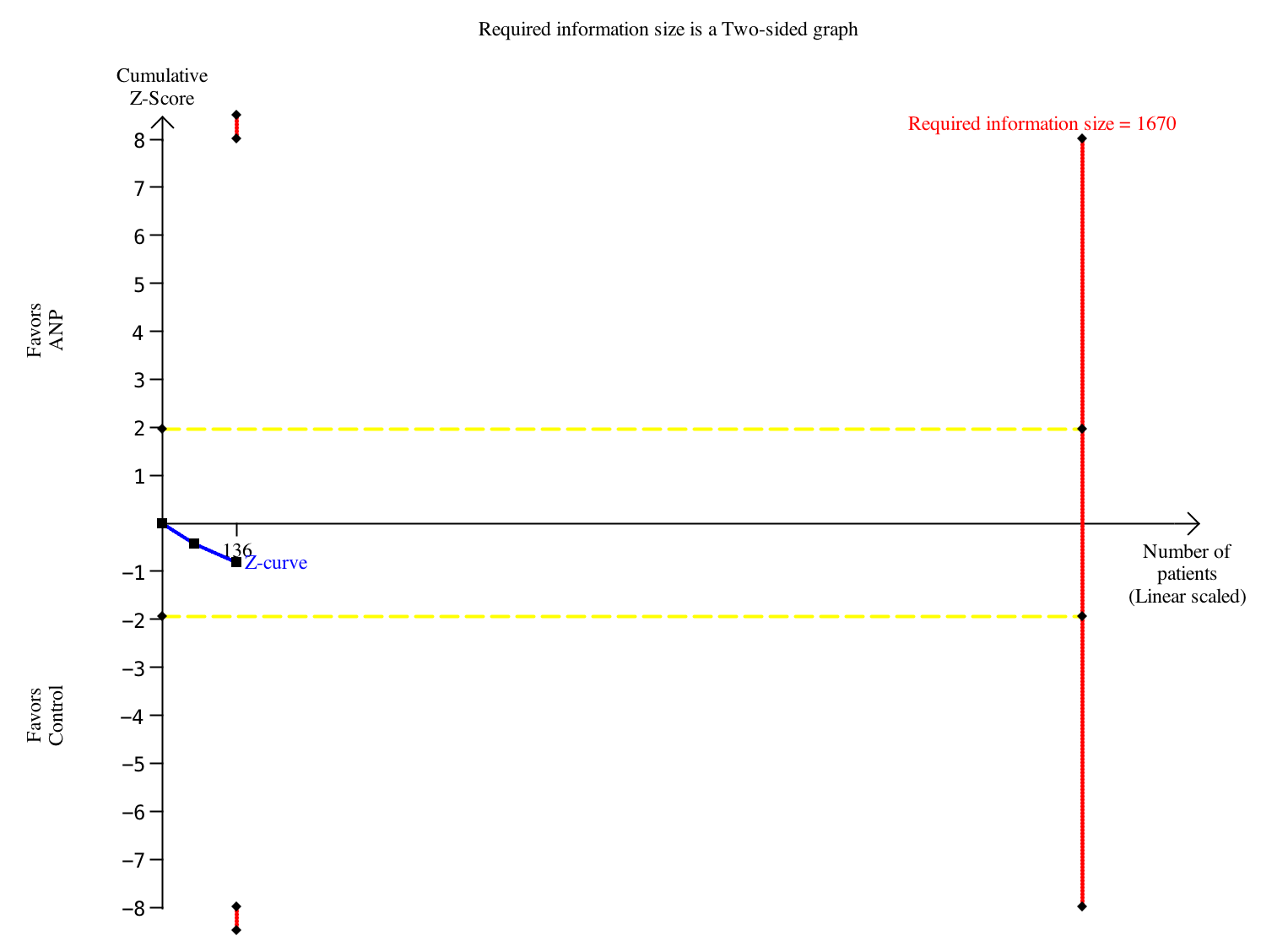
**

**Figure S15: Funnel plots in the prevention trials**

a. incidence of AKI, b. in-hospital mortality, c. renal replacement therapy, d. hospital stay, e. ICU stay, f. occurrence of hypotension, g. peak serum creatinine

**Figure S16: Funnel plots in the treatment trials**

a. renal replacement therapy, b. ICU stay, c. occurrence of hypotension

**Supplementary references**

1. Woolf AS, Mansell MA, Hoffbrand BI, Cohen SL, Moult PJ: The effects of low dose intravenous 99-126 atrial natriuretic factor infusion in patients with chronic renal failure. *Postgraduate medical journal* 1989, 65(764):362-366.

2. Kurnik BR, Weisberg LS, Cuttler IM, Kurnik PB: Effects of atrial natriuretic peptide versus mannitol on renal blood flow during radiocontrast infusion in chronic renal failure. *The Journal of laboratory and clinical medicine* 1990, 116(1):27-36.

3. Ratcliffe PJ, Richardson AJ, Kirby JE, Moyses C, Shelton JR, Morris PJ: Effect of intravenous infusion of atriopeptin 3 on immediate renal allograft function. *Kidney international* 1991, 39(1):164-168.

4. Sands JM, Neylan JF, Olson RA, O'Brien DP, Whelchel JD, Mitch WE: Atrial natriuretic factor does not improve the outcome of cadaveric renal transplantation. *Journal of the American Society of Nephrology : JASN* 1991, 1(9):1081-1086.

5. Lang CC, Henderson IS, Mactier R, Stewart WK, Struthers AD: Atrial natriuretic factor improves renal function and lowers systolic blood pressure in renal allograft recipients treated with cyclosporin A. *Journal of hypertension* 1992, 10(5):483-488.

6. Rahman SN, Kim GE, Mathew AS, Goldberg CA, Allgren R, Schrier RW, Conger JD: Effects of atrial natriuretic peptide in clinical acute renal failure. *Kidney international* 1994, 45(6):1731-1738.

7. Bergman A, Odar-Cederlof I, Westman L, Ohqvist G: Effects of human atrial natriuretic peptide in patients after coronary artery bypass surgery. *Journal of cardiothoracic and vascular anesthesia* 1996, 10(4):490-496.

8. Allgren RL, Marbury TC, Rahman SN, Weisberg LS, Fenves AZ, Lafayette RA, Sweet RM, Genter FC, Kurnik BR, Conger JD *et al*: Anaritide in acute tubular necrosis. Auriculin Anaritide Acute Renal Failure Study Group. *The New England journal of medicine* 1997, 336(12):828-834.

9. Lewis J, Salem MM, Chertow GM, Weisberg LS, McGrew F, Marbury TC, Allgren RL: Atrial natriuretic factor in oliguric acute renal failure. Anaritide Acute Renal Failure Study Group. *American journal of kidney diseases : the official journal of the National Kidney Foundation* 2000, 36(4):767-774.

10. Sezai A, Shiono M, Orime Y, Hata H, Hata M, Negishi N, Sezai Y: Low-dose continuous infusion of human atrial natriuretic peptide during and after cardiac surgery. *The Annals of thoracic surgery* 2000, 69(3):732-738.

11. Akamatsu N, Sugawara Y, Tamura S, Kaneko J, Togashi J, Kishi Y, Imamura H, Kokudo N, Makuuchi M: Prevention of renal impairment by continuous infusion of human atrial natriuretic peptide after liver transplantation. *Transplantation* 2005, 80(8):1093-1098.

12. Sezai A, Shiono M, Hata M, Iida M, Wakui S, Soeda M, Negishi N, Kasamaki Y, Saito S, Kato J *et al*: Efficacy of continuous low-dose human atrial natriuretic peptide given from the beginning of cardiopulmonary bypass for thoracic aortic surgery. *Surgery today* 2006, 36(6):508-514.

13. Sezai A, Hata M, Wakui S, Shiono M, Negishi N, Kasamaki Y, Saito S, Kato J, Minami K: Efficacy of low-dose continuous infusion of alpha-human atrial natriuretic peptide (hANP) during cardiac surgery: possibility of postoperative left ventricular remodeling effect. *Circulation journal : official journal of the Japanese Circulation Society* 2006, 70(11):1426-1431.

14. Sezai A, Hata M, Wakui S, Niino T, Takayama T, Hirayama A, Saito S, Minami K: Efficacy of continuous low-dose hANP administration in patients undergoing emergent coronary artery bypass grafting for acute coronary syndrome. *Circulation journal : official journal of the Japanese Circulation Society* 2007, 71(9):1401-1407.

15. Sezai A, Hata M, Niino T, Yoshitake I, Unosawa S, Wakui S, Fujita K, Takayama T, Kasamaki Y, Hirayama A *et al*: Continuous low-dose infusion of human atrial natriuretic peptide in patients with left ventricular dysfunction undergoing coronary artery bypass grafting: the NU-HIT (Nihon University working group study of low-dose Human ANP Infusion Therapy during cardiac surgery) for left ventricular dysfunction. *Journal of the American College of Cardiology* 2010, 55(17):1844-1851.

16. Yoshitake I, Sezai A, Hata M, Niino T, Unosawa S, Wakui S, Shiono M: Low-dose atrial natriuretic peptide for chronic kidney disease in coronary surgery. *Annals of thoracic and cardiovascular surgery : official journal of the Association of Thoracic and Cardiovascular Surgeons of Asia* 2011, 17(4):363-368.

17. Sezai A, Nakata K, Iida M, Yoshitake I, Wakui S, Hata H, Shiono M: Results of low-dose carperitide infusion in high-risk patients undergoing coronary artery bypass grafting. *The Annals of thoracic surgery* 2013, 96(1):119-126.

18. Sezai A, Nakata K, Iida M, Yoshitake I, Wakui S, Hata H, Shiono M: Early results of human atrial natriuretic peptide infusion in non-dialysis patients with chronic kidney disease undergoing isolated coronary artery bypass grafting: the NU-HIT trial for CKD-II. *Annals of thoracic and cardiovascular surgery : official journal of the Association of Thoracic and Cardiovascular Surgeons of Asia* 2014, 20(3):217-222.

19. Suzuki S, Yoshihisa A, Yamaki T, Sugimoto K, Kunii H, Nakazato K, Abe Y, Saito T, Ohwada T, Suzuki H *et al*: Long-term effects and prognosis in acute heart failure treated with tolvaptan: the AVCMA trial. *BioMed research international* 2014, 2014:704289.

20. Wang G, Wang P, Li Y, Liu W, Bai S, Zhen Y, Li D, Yang P, Chen Y, Hong L *et al*: Efficacy and Safety of 1-Hour Infusion of Recombinant Human Atrial Natriuretic Peptide in Patients With Acute Decompensated Heart Failure: A Phase III, Randomized, Double-Blind, Placebo-Controlled, Multicenter Trial. *Medicine* 2016, 95(9):e2947.

21. Takaya Y, Yoshihara F, Yokoyama H, Kanzaki H, Kitakaze M, Goto Y, Anzai T, Yasuda S, Ogawa H, Kawano Y *et al*: Impact of decreased serum albumin levels on acute kidney injury in patients with acute decompensated heart failure: a potential association of atrial natriuretic peptide. *Heart and vessels* 2017, 32(8):932-943.

22. Tsukamoto M, Koyama S, Esaki K, Hitosugi T, Yokoyama T: Low-dose carperitide (alpha-human A-type natriuretic peptide) alleviates hemoglobin concentration decrease during prolonged oral surgery: a randomized controlled study. *Journal of anesthesia* 2017, 31(3):325-329.

23. Kurnik BR, Allgren RL, Genter FC, Solomon RJ, Bates ER, Weisberg LS: Prospective study of atrial natriuretic peptide for the prevention of radiocontrast-induced nephropathy. *American journal of kidney diseases : the official journal of the National Kidney Foundation* 1998, 31(4):674-680.

24. Morikawa S, Sone T, Tsuboi H, Mukawa H, Morishima I, Uesugi M, Morita Y, Numaguchi Y, Okumura K, Murohara T: Renal protective effects and the prevention of contrast-induced nephropathy by atrial natriuretic peptide. *Journal of the American College of Cardiology* 2009, 53(12):1040-1046.

25. Sezai A, Hata M, Niino T, Yoshitake I, Unosawa S, Wakui S, Osaka S, Takayama T, Kasamaki Y, Hirayama A *et al*: Influence of continuous infusion of low-dose human atrial natriuretic peptide on renal function during cardiac surgery: a randomized controlled study. *Journal of the American College of Cardiology* 2009, 54(12):1058-1064.

26. Tamura Y, Nagata H, Sato Y, Nitta H, Wakabayashi G: [Usefulness of human atrial natriuretic peptide (hANP) on perioperative management for liver resection]. *Masui The Japanese journal of anesthesiology* 2011, 60(3):343-352.

27. Sezai A, Hata M, Niino T, Yoshitake I, Unosawa S, Wakui S, Kimura H, Shiono M, Takayama T, Hirayama A: Results of low-dose human atrial natriuretic peptide infusion in nondialysis patients with chronic kidney disease undergoing coronary artery bypass grafting: the NU-HIT (Nihon University working group study of low-dose HANP Infusion Therapy during cardiac surgery) trial for CKD. *Journal of the American College of Cardiology* 2011, 58(9):897-903.

28. Okumura N, Hayashi M, Imai E, Ishii H, Yoshikawa D, Yasuda Y, Goto M, Matsuo S, Oiso Y, Murohara T: Effects of carperitide on contrast-induced acute kidney injury with a minimum volume of contrast in chronic kidney disease patients. *Nephron extra* 2012, 2(1):303-310.

29. Mori Y, Kamada T, Ochiai R: Reduction in the incidence of acute kidney injury after aortic arch surgery with low-dose atrial natriuretic peptide: a randomised controlled trial. *European journal of anaesthesiology* 2014, 31(7):381-387.

30. Moriyama T, Hagihara S, Shiramomo T, Nagaoka M, Iwakawa S, Kanmura Y: The protective effect of human atrial natriuretic peptide on renal damage during cardiac surgery. *Journal of anesthesia* 2017, 31(2):163-169.

31. Mitaka C, Kudo T, Jibiki M, Sugano N, Inoue Y, Makita K, Imai T: Effects of human atrial natriuretic peptide on renal function in patients undergoing abdominal aortic aneurysm repair. *Critical care medicine* 2008, 36(3):745-751.
